# Predicting individual brain maturity using dynamic functional connectivity

**DOI:** 10.3389/fnhum.2015.00418

**Published:** 2015-07-16

**Authors:** Jian Qin, Shan-Guang Chen, Dewen Hu, Ling-Li Zeng, Yi-Ming Fan, Xiao-Ping Chen, Hui Shen

**Affiliations:** ^1^College of Mechatronics and Automation, National University of Defense Technology, ChangshaChina; ^2^National Key Laboratory of Human Factors Engineering, China Astronaut Research and Training Center, BeijingChina

**Keywords:** development, functional connectivity, fMRI, multivariate pattern analysis, low-frequency fluctuation

## Abstract

Neuroimaging-based functional connectivity (FC) analyses have revealed significant developmental trends in specific intrinsic connectivity networks linked to cognitive and behavioral maturation. However, knowledge of how brain functional maturation is associated with FC dynamics at rest is limited. Here, we examined age-related differences in the temporal variability of FC dynamics with data publicly released by the Nathan Kline Institute (NKI; *n* = 183, ages 7–30) and showed that dynamic inter-region interactions can be used to accurately predict individual brain maturity across development. Furthermore, we identified a significant age-dependent trend underlying dynamic inter-network FC, including increasing variability of the connections between the visual network, default mode network (DMN) and cerebellum as well as within the cerebellum and DMN and decreasing variability within the cerebellum and between the cerebellum and DMN as well as the cingulo-opercular network. Overall, the results suggested significant developmental changes in dynamic inter-network interaction, which may shed new light on the functional organization of typical developmental brains.

## Introduction

Typical brain development, as a prerequisite for studying developmental disorders and pediatric-onset neuropsychiatric diseases, has received increasing attention in recent years (Shaw et al., 2008; Westlye et al., 2010). Increasing numbers of functional magnetic resonance imaging (fMRI)-based functional connectivity (FC) analyses have revealed significant changes in inter-regional interactions over brain development in several intrinsic connectivity networks (ICNs), including the prefrontal, sensorimotor, salience, and default mode networks (DMNs). In particular, specific developmental trends, such as the strengthening of long-range connections and weakening of short-range connections (Dosenbach et al., 2010), the strengthening of temporal segregation between task-positive and DMNs (Thomason et al., 2008), and a shift in the locations of cortical hubs from primary sensory and motor regions to heteromodal association cortex (Fransson et al., 2011), have been suggested to underlie the improvements in cognitive ability and emotional processing that occur during maturation (Dosenbach et al., 2010; Betzel et al., 2014). Thus, the investigation of age-related differences in the spatiotemporal properties of whole-brain FC is fundamentally important for the understanding of developmental features in brain functional organization and the development of feasible markers of developmental trajectories.

Although FC studies have documented reliable changes in human functional brain maturity throughout development, temporal stationarity was typically assumed in previous studies, that is, that FC could be measured over the entire fMRI scan. However, recent neuroscience studies have suggested that resting-state FC is dynamic and exhibits significant spontaneous fluctuation (Chang and Glover, 2010; Jones et al., 2012; Hutchison et al., 2013a), which has also been observed in the anesthetized macaque brain (Hutchison et al., 2013b) and verified by electrophysiological [electroencephalography (EEG)] data (Von Der Malsburg et al., 2010). Furthermore, brain dynamics related to temporal variations in large-scale topological properties have also been reported based on both high-resolution resting-state fMRI (rs-fMRI) data from human brains and simulated rs-fMRI data from macaques (Zalesky et al., 2014). It is notable that the low-frequency fluctuations observed in resting-state FC exhibit complex spatiotemporal structures, which can be further identified as multiple discrete, reproducible patterns (Hutchison et al., 2013a; Allen et al., 2014; Yang et al., 2014). Thus, resting-state FC dynamics have been suggested to reflect ongoing dynamic interactions across distributed regions associated with cognitive and behavioral abilities (Hutchison et al., 2013a). For example, some state-specific temporal features, such as the dwell time of select states, change with age although the brain’s repertoire of functional states is generally preserved (Hutchison and Morton, 2015). During the transition from childhood to adulthood, the brain undergoes ongoing functional reorganization that impacts cognition and behavioral abilities. Hence, we predict that the identification of age-related changes in the temporal attributes of dynamic inter-regional interactions (functional connections) at rest will elucidate fundamentally important patterns of functional reorganization, which are linked to cognitive and behavioral maturation. To date, however, there is very limited knowledge regarding how functional brain maturation is associated with FC dynamics at rest.

The goal of this study was to test the impact of brain maturation on the temporal variability of FC fluctuations across sliding time windows. An increasing number of studies have suggested that low-frequency oscillations in resting-state FC are linked to spontaneous shifts between various forms of conscious brain processing, such as passive mind wandering, active monitoring, memory formation, or changes in attention and arousal during image acquisition (Hutchison et al., 2013b; Liu and Duyn, 2013). Hence, it is reasonable that the temporal variability of spontaneous fluctuation in FC may contain specific information on processing patterns and capacity of functional brain systems, which may change with age during maturation. This idea is also inspired by recent findings on the association between variability in dynamic FC and behavior. For instance, the individual differences in variability of FC exhibit significant correlation with the tendency to attend to pain (Kucyi et al., 2013) and are related to the degree to which a subject is mind-wandering away from a sensory stimulus (Kucyi and Davis, 2014). Disease-related alterations in the dynamic properties of FC have also been reported, suggesting that temporal features of FC associated with the cognitive dysfunction in diseases can potentially serve as disease biomarkers (Jones et al., 2012; Damaraju et al., 2014; Shen et al., 2014). More importantly, cognitive information processing, especially high-order cognitive function of human brains arises from specific patterns of spatiotemporal activity within networks and functional interaction across distinct networks. Increasing age has been observed to be linked to greater variability in connection strength across time at rest, while less variability has been observed among older participants compared to younger participants during the administration of a cognitive control task (Hutchison and Morton, 2015).

We used the amplitude of low-frequency fluctuation of FC (ALFF-FC) as a metric to measure temporal variability of spontaneous fluctuation in resting-state FC. The ALFF-FC is a simple but appropriate measure of signal variability that is defined as the total signal power within the low-frequency range (Han et al., 2011; Yu et al., 2014). We predicted that the fluctuation of correlations across ICNs, which reflects the frequency of inter-network interactions and communication, would vary by age. Further, we used a predictive model to test this hypothesis by asking whether the temporal property of dynamic FC can be used to sufficiently predict brain maturity at an individual level. Compared to group-level fMRI studies, the use of predictive model to make continuously values predictions may have implications in clinical scenarios for developmental disorders. It has been suggested that multivariate pattern analysis can extract sufficient information from rs-fMRI-based FC to make accurate predictions regarding an individual’s brain maturity across development (Dosenbach et al., 2010). During brain development from childhood to senescence, functional connections tend to increase linearly in the emotional system and decrease in the sensorimotor system, whereas quadratic trajectories have been observed in the functional connections associated with higher-order cognitive functions (Wang et al., 2012b). Here, we extend these findings to dynamic aspects of FC. In particular, we hope to identify the relevant FC with significant developmental trends that could further our understanding of typical functional brain development.

## Materials and Methods

### Participants and fMRI Data Acquisition

Resting-state fMRI data were collected from 183 healthy, the participants (age range, 7–30 years; mean age, 19.9 ± 5.3 years; 84 males) from the NKI/Rockland Sample (NKI-RS), which is provided by the Nathan Kline Institute (NKI, Orangeburg, NY, USA) and is available online in a public database^1^ (Nooner et al., 2012). All approvals and procedures for collection and sharing of data were approved by the NKI institutional review board, and each participant gave written informed consent. For children who were unable to give informed consent, written informed consent was obtained from their legal guardians. A detailed distribution of age and sex of the whole subjects is shown in Supplementary Figure S1. There are several papers published only based upon a subset of NKI-RS sample (Uddin, 2011; Oler, 2012; Laird et al., 2013; Betzel et al., 2014) for controls in developmental or adulthood studies.

Magnetic resonance imaging (MRI) data were acquired on a 3.0 T SIMENS Trio scanner. Each subject underwent a 10-min or resting-state fMRI scan involving an echo-planar imaging (EPI) sequence. 85 subjects of the total healthy from NKI were scanned by following parameters: TR/TE = 2500/30 ms, FA = 80, FOV = 216 mm, matrix = 64 × 64, slices = 38, thickness = 3.0 mm and time points = 260 and the other subjects were scanned by following parameters: TR/TE = 645/30 ms, slices = 40, thickness = 3.0 mm and time points = 900. For each subject, high-resolution T1-weighted images were also acquired using the magnetization-prepared rapid gradient echo (MPRAGE) sequence with the parameters of TR/TE = 2500/3.5 ms, FA = 8, thickness = 1.0 mm, slices = 192, matrix = 256 × 256, and FOV = 256 mm). The quality of the fMRI data was good, and the head motion of all of the subjects was small (head motion < 1 mm and rotation < 1°).

### Data Preprocessing

For the functional images, slice-time correction per volume and head motion correction per run were performed using the statistical parametric mapping software package SPM8^2^. Each volume of functional images was registered to the initial volume within the run; then, the mean volume of each run was generated by averaging all of the volumes resliced to the first volume. In addition, we estimated six head motion parameters with registration for further data preprocessing. During each run, the mean functional image was registered to the participant’s structural image using a rigid body transformation model, and the structural image was registered into the standard T1 template in the Montreal Neurological Institute (MNI) space using a non-linear transformation algorithm with FSL software^3^. To a certain degree, performing non-linear transformation between the structural image and standard template can reduce the effects of anatomical differences on maturation and aging of the brain (Garrett et al., 2010). The above data preprocessing yielded, three image transformation models: the transformation from each volume to the initial volume within the run, the transformation from the mean functional volume to the structural volume, and the transformation from the individual structural volume to the standard MNI structural template. By concatenating the above three transformations sequentially, we obtained a direct transformation from each initial functional volume to the standard MNI space, allowing each initial functional image to be directly normalized into the standard MNI space and resliced to 3 mm × 3 mm × 3 mm. Then, the normalized functional volumes were spatially smoothed using a Gaussian filter kernel with 6 mm FWHM. Temporal band-pass filtering from 0.01 to 0.08 Hz was implemented on the smoothed fMRI series to remove the signals from other frequency bands. Linear detrending processing was used to remove the linear signal drift. By averaging the unsmoothed fMRI time series of voxels within the whole brain, white matter (WM), and a ventricular region of interest, separately, we obtained the whole brain, WM, and cerebrospinal fluid (CSF) signals. Prior to the nuisance regression, these nuisance signals as regressors were band-pass filtered to the same frequency range as the fMRI time series to avoid reintroducing unwanted frequency content (Hallquist et al., 2013). To further reduce signal noise, the fMRI series were corrected by regressing with the head motion parameters and the WM, CSF, and whole-brain signals (Zeng et al., 2012). The residual of regression was used for further processing. Importantly, all of the subjects were later divided into two groups according to head motion and TR for the control analyses of movement and data type, respectively.

### ALFF-FC Map of Dynamic Functional Connectivity

Regions of interest (ROI)-based brain signals were generated by averaging the regressed fMRI series of voxels within each gray matter region (Shen et al., 2010) according to the AAL atlas, which anatomically divides the human brain cortex into 116 regions, including 90 cerebrum regions and 26 cerebellum regions. The AAL atlas masks were obtained using the WFU_PickAtlas software package^4^. Then, the inter-regional dynamic FC network was captured using Pearson’s temporal correlation of each region signal pair within a sliding time window size of 36 s. We ultimately obtained a series of 116 × 116 connectivity matrices in which each element reflects the correlation coefficient of corresponding connections within a particular sliding window. To normalize the correlation coefficient values, Fisher’s *z*-transform was performed on each connectivity matrix. To avoid repeated information, only the upper triangular portion of the symmetrical FC matrix was properly reformed into a correlation coefficient vector for further analysis.

The fluctuation amplitude of the correlation coefficient time courses represents the variability of each connection between regions over time. Previous studies have demonstrated that the ALFF-FC is an appropriate measure of signal fluctuation, which is defined as the total signal power within the low-frequency range (Han et al., 2011; Yu et al., 2014). However, the sliding window method may lead to the emergence of spurious fluctuations in sliding-window correlation due to a mismatch between the choice of the window length and high-pass filtering of the original time courses (Hutchison et al., 2013a; Leonardi and Van De Ville, 2015). A high-pass filtering for the original BOLD signals and a low-pass filtering for correlation coefficient time series with the cut-off frequency 1/*w* are suggested to remove spurious fluctuations in dynamic FC, when a certain window size *w* is given (Leonardi and Van De Ville, 2015). Hence, for a given window size *w*, we high-pass filtered the ROI signals with cut-off frequency 1/*w* prior to calculation of connectivity matrices, and then low-pass filtered the correlation coefficient time series with cut-off frequency 1/*w*. The cut-off frequency and the number of data points for each window size are listed in Supplementary Table S1. Accordingly, ALFF-FC values were calculated within the frequency band of dynamic FC from 0 to 1/*w*. The fast Fourier transform (FFT) was applied to the correlation coefficient time series of each dynamic FC; then, the ALFF-FC index was calculated by summing the FFT coefficients within the frequency band range 0 to 1/*w*. The ALFF-FC map of the correlation coefficient vector, which measures the variability of the whole-brain dynamic FC network, was obtained as a high dimensional feature for subsequent prediction of the brain maturity of each subject.

### Partial Least-Squares Analysis

To investigate variability in the development of dynamic FC networks during maturation, partial least-squares analysis (PLS), which evaluates the relationship between ALFF-FC maps of the dynamic FC network and maturation age, was performed on the total of subjects (aged 7–30 years). PLS is a multivariate analysis method that is normally used to capture the multivariate patterns of brain structure (Kauffmann et al., 2015) or functional activity (McIntosh et al., 2014). In this analysis, the correlation coefficient matrix of age and the ALFF-FC of each dynamic FC were first calculated across subjects. Then, latent variables (i.e., the singular value) and so-called “connection saliences” (age-related weights across connections) were obtained using singular value decomposition (SVD) of the correlation coefficient matrix described above. Latent variables indicate the correlation strength, and connectivity salience reflects the correlation salience of each dynamic FC with age on corresponding latent variables. Due to the simplicity of this analysis (age is the only behavioral variable), there is one latent dimension for PLS analysis, which allows the connectivity salience to directly reflect the correlation between each dynamic FC and age. Using the dot product of connectivity saliences and the ALFF-FC coefficient vector, we obtained brain maturation scores, a synthetic measure of the age-dependent pattern of the dynamic FC network on the corresponding latent variables.

To evaluate the significance of the latent variables, 1000 permutation tests were performed on singular values, by sampling without replacement and repeated PLS analysis on permutation samples. Then, the reliability of the connectivity salience was assessed using 1000 bootstrapping tests with resampling and data replacement. The bootstrap ratio, a normalized measure of the reliability of the connectivity salience, was calculated by dividing the bootstrap mean salience of each connection by its standard error. Finally, connections with a bootstrap ratio value that exceeded 3.0 (∼99% confidence interval) were selected as age-dependent “predictive connections.”

### Predicting Age from Brain Maturation Scores

**FIGURE 1:**
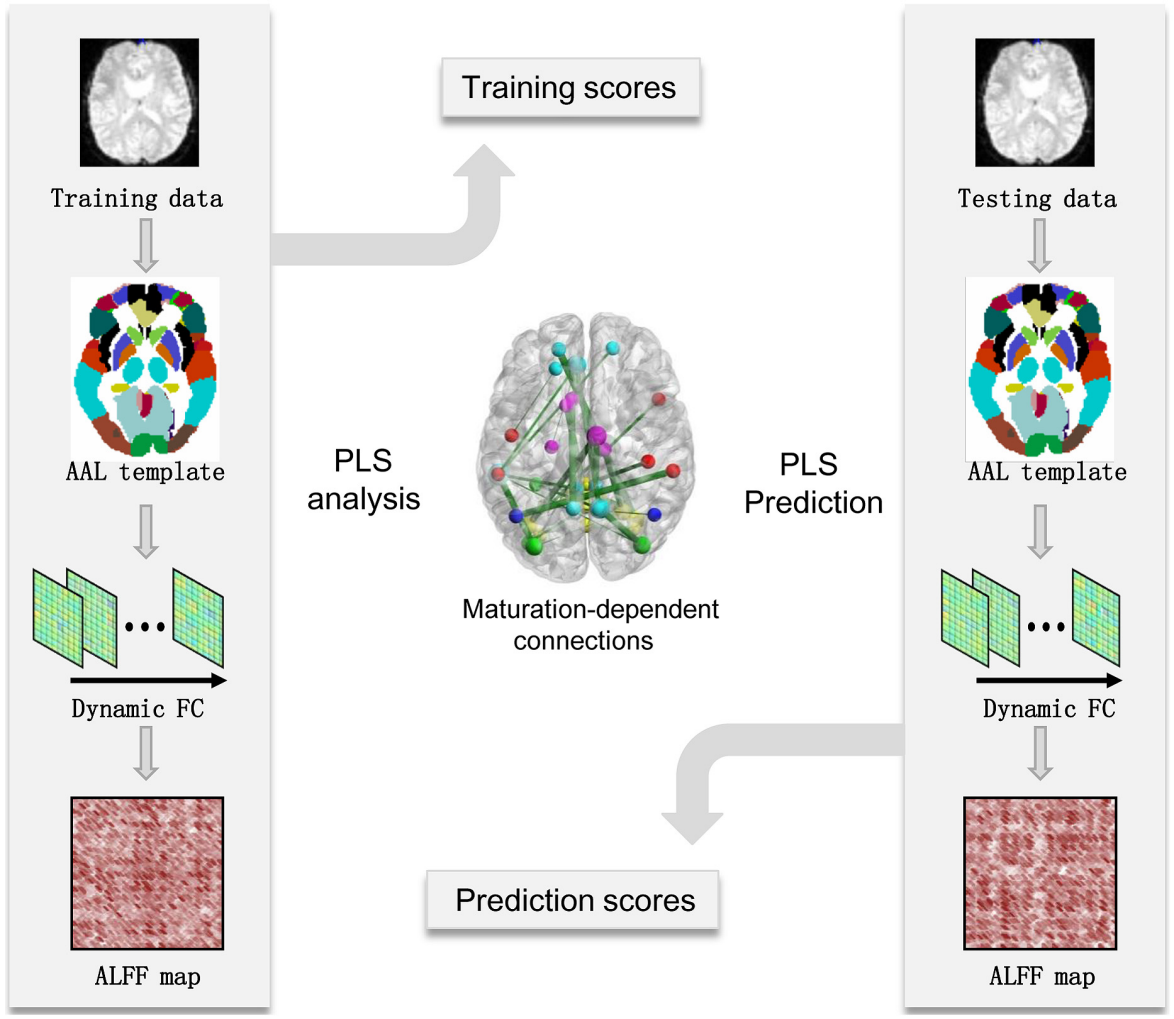
**Flowchart of maturity prediction using the dynamics of resting-state functional connectivity.** AAL, automated anatomical labeling; ALFF-FC, amplitude of low-frequency fluctuation of functional connectivity; FC, functional connectivity; PLS, partial least-squares.

Brain maturation scores offered an estimation of age-dependent patterns in the dynamic FC network, which can be used to predict the chronological age of the brain. For subjects in the maturation group, PLS regression models were used to predict chronological age. To measure the performance of the PLS regression model, the mean absolute error (MAE) of age prediction was calculated using the leave-one-out cross-validation (LOOCV) method. A predictable regression model may indicate significant and reliable age-dependent patterns in the dynamic FC networks captured using the PLS analysis (see **Figure 1** for flowchart).

### Region Weight for Regions of Interest (ROIs)

We computed the “region weight” for each ROI, which represents the relative contribution of the various ROIs to brain maturity prediction (Dosenbach et al., 2010). The weight of each region was obtained by summing all bootstrap ratios of predictive connections associated with this region. A region with a weight of zero was assigned to ROIs which that did not form any “predictive functional connections.” To visually represent the relative contribution of individual ROIs to functional brain maturity prediction, the diameters of the spheres representing the ROIs were scaled to the region strengths of the ROIs.

### Control Analysis

Choosing an appropriate window size is an area of concern when using the sliding window approach to estimate FC dynamics. Theoretically, the window size should be sufficiently small enough to detect potentially interesting transients in the low-frequency fluctuations in FC (Sakoğlu et al., 2010; Hutchison et al., 2013b). However, an excessively small window will decrease the signal to noise ratio (SNR) of the estimated FC due to a smaller number of time points available and increases in the higher frequencies of the fMRI time series (Hutchison et al., 2013b). It has been suggested that cognitive states may be correctly identified from covariance matrices estimated from as little as 30–60 s of data (Shirer et al., 2012). Here, we investigated the stability of identified age-dependent dynamic FC features and the potential impact of window sizes that varied from 20 to 80 s on maturity prediction performance.

Additionally, the influence of head motion on the observed dynamics should be considered, even though we have attempted to minimize its impact with linear regression and low-frequency filtering during the preprocessing step (Kucyi and Davis, 2014). Head motion has been showed to have significant, systematic effects on FC MRI network measures, especially in the default and fronto-parietal control networks (Van Dijk et al., 2012), suggesting the need for greater care when dealing with previous findings regarding brain development (Kucyi and Davis, 2014). Furthermore, a recent study has expanded our understanding of head motion in brain imaging by showing the significant neurobiological basis of head motion in brain imaging (Zeng et al., 2014). Here, we computed the average amplitude of head motion within each sliding window or over the entire scan as follows (Wang et al., 2012a):

$$Translation/Rotation\,\;=\;\frac{1}{n-1}\overset{n}{\underset{i=2}{\Sigma }}\sqrt{{\left|x_{i}-x_{i-1}\right|}^{2}+{\left|y_{i}-y_{i-1}\right|}^{2}+{\left|z_{i}-z_{i-1}\right|}^{2}}$$

where *n* is the number of points in the time series and x_i_,y_i_, and z_i_ are the translations/rotations at the *i*^th^ time point in the x,y, and z directions, respectively. We evaluated the potential impact of head motion on the temporal property of resting-state FC using two methods. In the first method, we regressed out the average head-motion signal as confounds within each sliding window from the time series of sliced FC. In the second method, for each subject, we regressed out the mean head motion over the entire scan from the ALFF-FC values of each connection, to exclude the possible correlation between average head motion and age. Finally, for both of regression models, we found no significant differences between the results of performance prediction with and without motion regression (Supplementary Figure S5). In addition, to determine whether head motion and FC dynamics were significantly related, we also calculated the correlation between mean head motion and ALFF-FC for each connection across subjects.

The correlation of head movement with age (*R* = -0.221, *P* < 0.005; see Supplementary Figure S6A) were found in present subjects. To make a better control for head movement, we also selected 103 subjects whose age is uncorrelated with movement (*P* > 0.9; see Supplementary Figure S6C). We repeated the analysis on the subset of subjects without significant differences in head motion, and obtained the similar results of significant correlation between brain scores of dynamic FC and maturation ages (*R* = 0.797, *P* < 0.0001; see Supplementary Figures S6B,D), demonstrating that our results are motion-independent.

Signal dropout and image artifacts may also affect the results. Thus, we calculated a temporal SNR map of each subject’s motion-corrected fMRI data. The SNR map was calculated for each voxel by averaging the signal intensity across the whole run and dividing it by the standard deviation over time (Buckner et al., 2011). In addition, all of the subjects were divided into a young group (7–20 years, 89 subjects) and an adult group (20–30 years, 94 subjects). The average SNR maps of the two groups were calculated (see Supplementary Figure S7). To identify regions with an SNR that differed significantly between the two age groups, the SNR maps of the groups were compared with a two-sample *t*-test (*P* < 0.05, FDR corrected). In addition, we regressed out the temporal SNR (Van Dijk et al., 2012) from the ALFF-FC value of each connection across subjects and repeated the PLS analyses to further exclude the effect of artifacts.

For estimating to reproducibility, the subjects from NKI were divided into the two groups (the first group: 85 subjects, TR = 2500 ms; the second group: 98 subjects, TR = 645 ms) according to the TR of parameter in their fMRI scaning. The same PLS analyses were also performed on the each separate group with the same window size of 36 s, respectively. Note that the experiment parameters between the groups such as the repetition time are different, such that the potential impact of different repetition time and individual variability on main conclusions of this research can be evaluated.

## Results

### Age-Dependent Changes in the Variability of the Dynamic FC during Maturation

**FIGURE 2:**
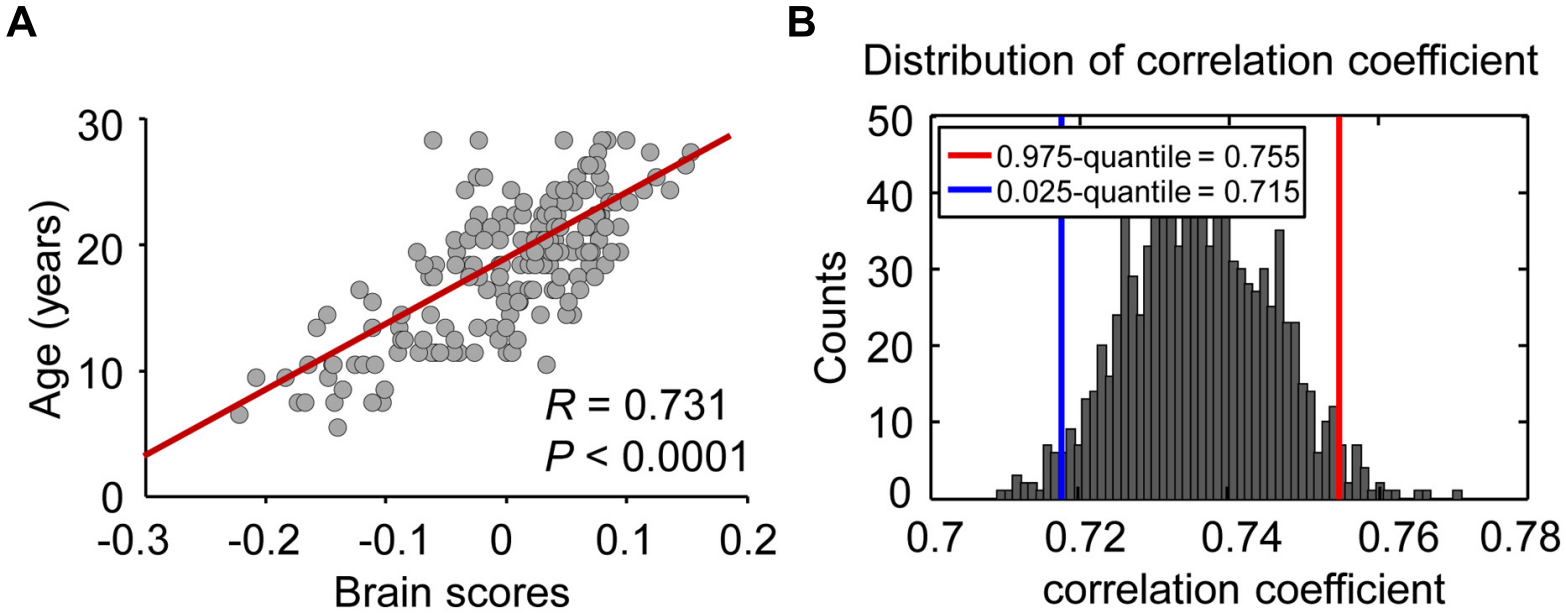
**Relationship between brain score and maturation age.(A)** Individual brain scores, reflecting the maturity of the brain, were equal to the dot products of connectivity salience and ALFF-FC coefficient vectors. Scatter plots of the age and brain score were constructed to view the correlation between these two parameters, and age was treated as a continuous variable. **(B)** In the reliability test, 1000 bootstrap resamples were performed to estimate the distribution and 95% confidence intervals of correlation coefficients between brain score and age.

**Figure 2** demonstrates a strong correlation (*R* = 0.731, permuted *P* < 0.02) between brain scores from the PLS analysis and chronological age, suggesting a significant correlation between variability in the dynamic FC and age during maturation. To evaluate the reliability of the correlation, a bootstrapping test (1,000 times) was performed on all subjects, and the bootstrapped confidence interval (CI; 95% CI for *R* = 0.715, 0.755) was estimated. Connections with a bootstrap ratio over 3.0 were considered to be significantly correlated with age.

**FIGURE 3:**
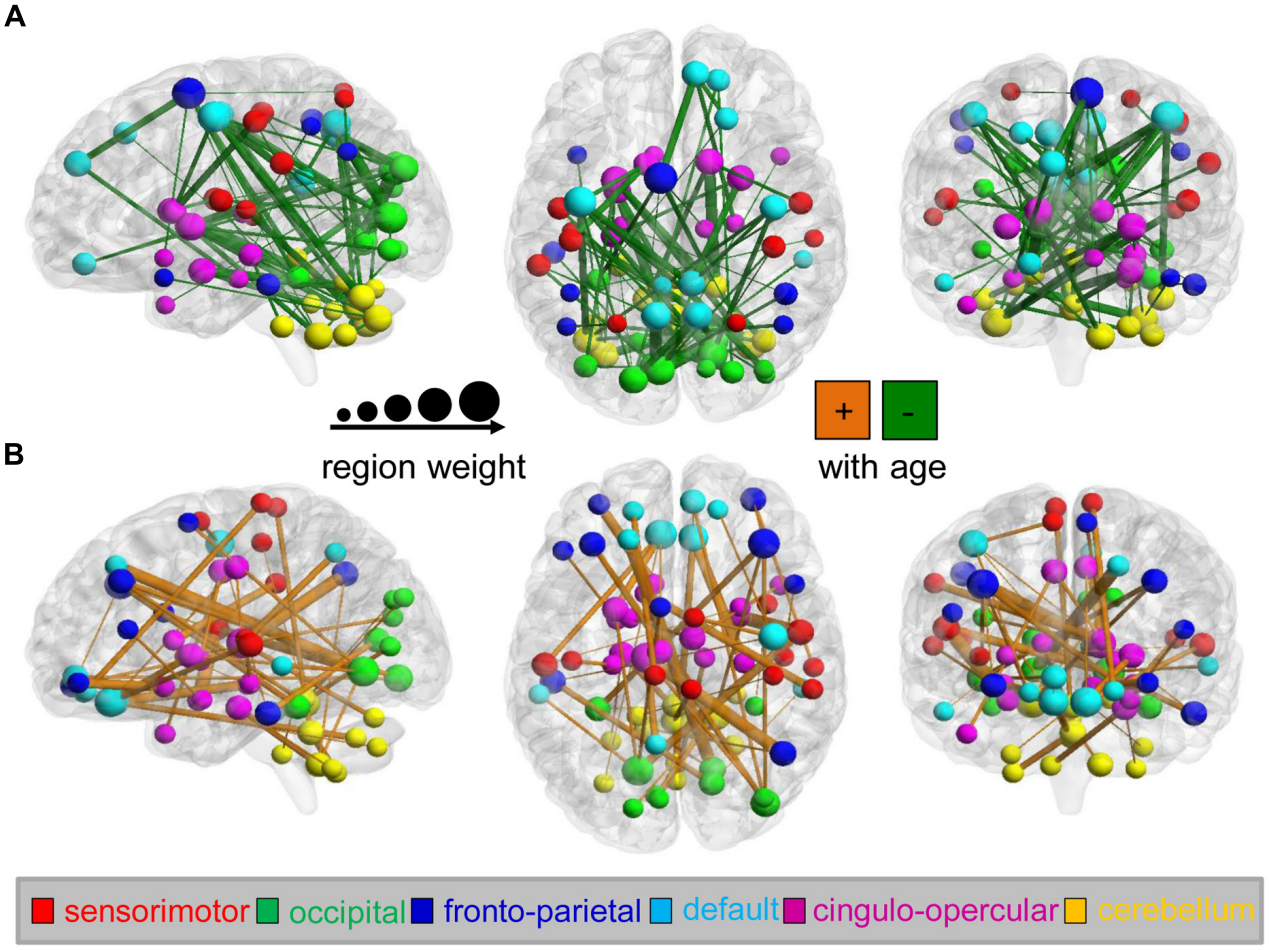
**Maturation-dependent connections and regions in the dynamics of functional connectivity from the analysis on the total of subjects.** Only connections with bootstrap ratios over 3.0 were selected and considered predictive connections. **(A)** Functional connections that decreased with maturation during the PLS analysis (shown in green) and maturation-dependent ROIs are displayed on the surface of the brain. Connection lines are scaled according to the bootstrap ratios. The color and scale of ROIs represent the functional networks and region weights (1/2 the sum of connection bootstrap ratios to and from that region). In contrast, **(B)** functional connections that increased with maturation are displayed in orange.

Among the identified predictive connections, we found both increasing and decreasing trends in the variability of the dynamic FC with maturation age (**Figure 3**). However, connections with various developmental trends regarding maturation age indicate distinct distributions in the six functional networks (the sensorimotor, occipital, fronto-parietal, and DMN; the cingulo-opercular network (CON); and the cerebellum). Network affiliations among the 116 ROIs according to the AAL template were provided by He et al. (2009). An increasing trend with age was mainly found in connections between the cerebellum and occipital network (e.g., the superior occipital gyrus, lingual gyrus, calcarine fissure, and surrouding cortex) and DMN (e.g., the medial superior and orbital middle frontal gyrus, the posterior cingulate gyrus and inferior temporal gyrus), as well as within the cerebellum and DMN, as shown in **Figure 3A**. In contrast, a decreasing trend was found in connections within the cerebellum and connections between the cerebellum and DMN (e.g., the precuneus, posterior cingulate, anterior cingulate and paracingulate gyri, and the orbital middle frontal gyrus) and CON (e.g., the caudate nucleus, middle temporal pole, median cingulate and paracingulate gyri), as shown in **Figure 3B**. Bootstrap ratios and region labels of each predictive connection are listed in Supplementary Tables S2 and S3, and the overall distribution of bootstrap ratios of the connections shown in Supplementary Figure S2. In the PLS analysis on each maturation group, age-dependent patterns of connectivity are similar to that in analysis on the total of subjects, as shown in Supplementary Figure S3.

**FIGURE 4:**
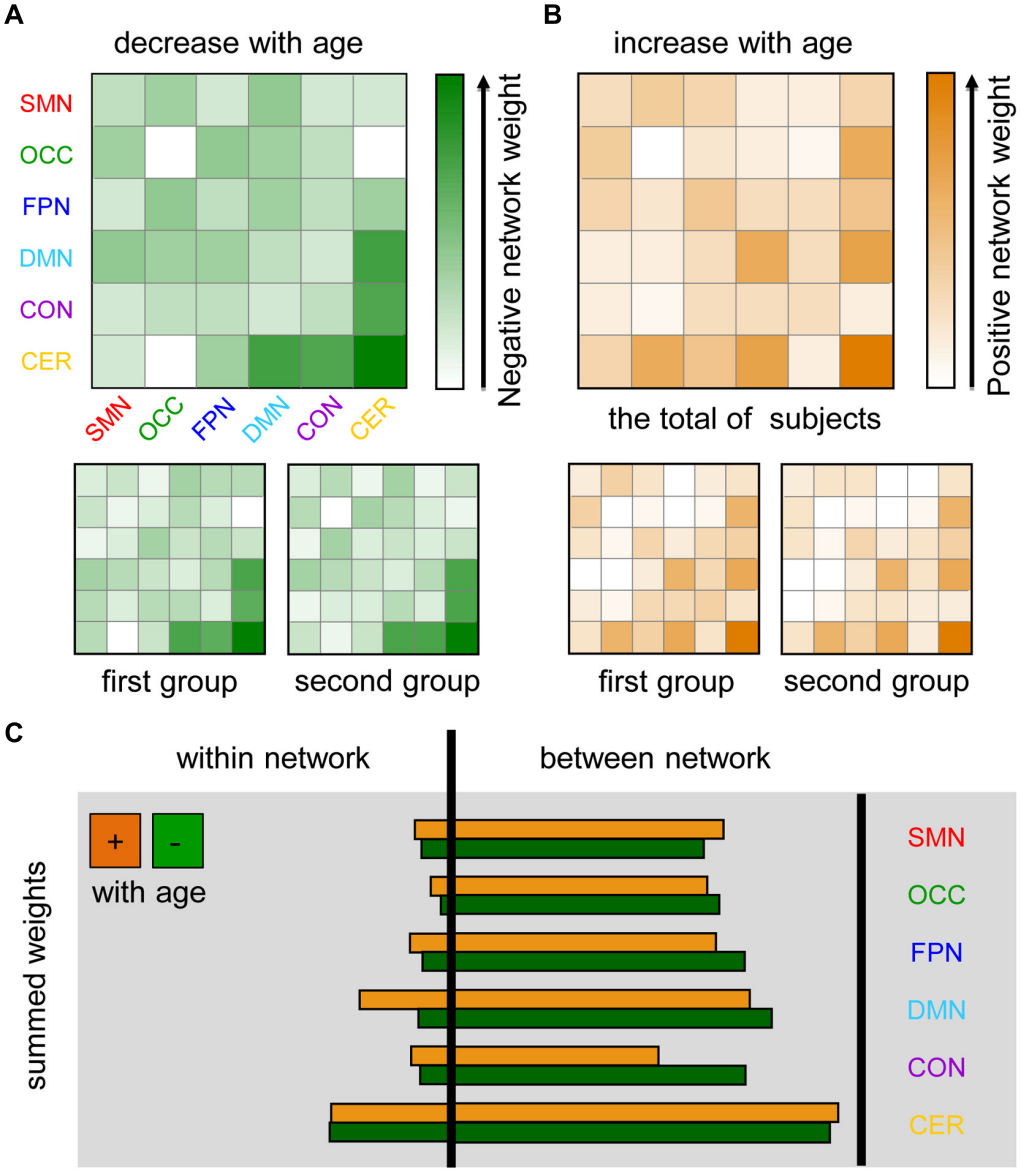
**Maturation-related networks in the dynamics of functional connectivity.** SMN, sensorimotor network; OCC, occipital network; FPN, fronto-parietal network; DMN, default mode network; CON, cingulo-opercular network; CER, cerebellum. The first group includes 85 subjects with TR = 2500 ms and the second group contains 98 subjects with TR = 645 ms. **(A)** Negative network weight (1/2 the sum of the bootstrap ratios of connections that decreased with age affiliated with corresponding networks) reflects the “network-distribution” of connections that decrease with age in dynamics. **(B)** The positive network weight, which was calculated by a similar method, represents the distribution of connections that increase with age. **(C)** The sum of all of the connection bootstrap ratios within each network and between networks are shown to the left and right of the black line, respectively.

To further evaluate the distribution of age-dependent connectivity in networks, a “network bootstrap ratios” matrix was calculated in which each element is equal to the sum of reliable connection bootstrap ratios within or between associated networks (**Figure 4**). **Figures 4A,B** indicate the network distribution of the respective decreased and increased connections with maturation age. Both in the analyses on each group and the total of subjects, network bootstrap ratios matrices are reproducible. Furthermore, age-dependent connections were mainly located between functional networks, except for a portion of age-dependent connections located within the cerebellum and DMN (**Figure 4C**).

### Age Prediction Performance

Brain scores reflect the development of a participant’s age-related brain connections and are strongly correlated with chronological age (**Figure 2**). We used a PLS regression mode to predict maturation age. To measure the performance of the PLS-based age prediction mode, the prediction error (for the total of subjects, MAE = 4.6 years, SE = 0.3 years; for the first group, MAE = 4.7 years, SE = 0.3 years; for the second group, MAE = 4.8 years, SE = 0.4 years) between predicted age and chronological age was calculated using a LOOCV method. The accurate and reliable age prediction demonstrated not only the presence of a relationship between maturation age and the dynamics of FC, but also a strong relationship between the connections in spatial patterns captured from PLS analyses and maturation age.

### Influence of the Sliding Time Window Size

**FIGURE 5:**
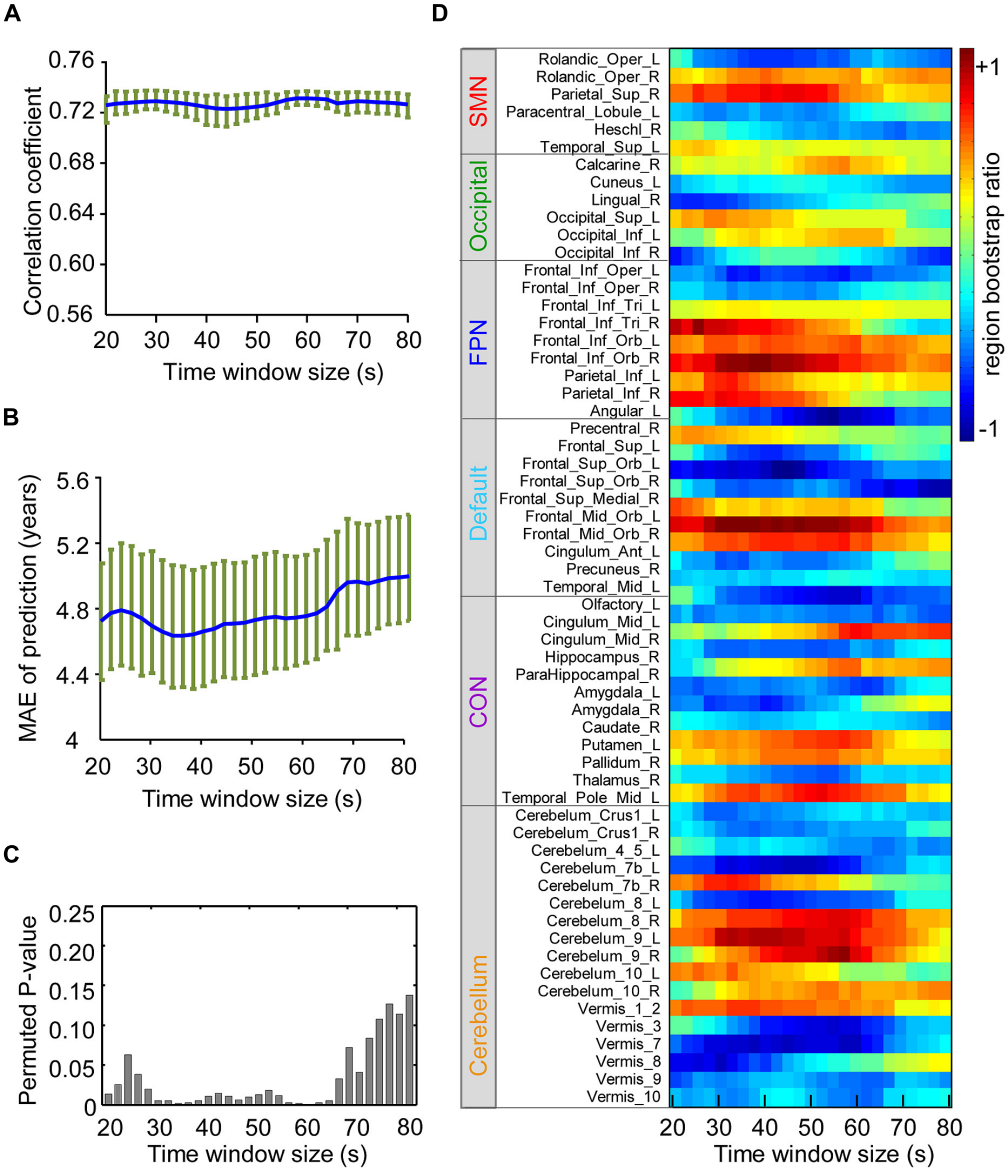
**Influence of the sliding windows size on the results of partial least-squares analysis.** SMN, sensorimotor network; OCC, occipital network; FPN, fronto-parietal network; **(A)** Correlation coefficients of the brain score and age with each time window size are shown in blue, and the green circle above indicate the confidence intervals. **(B)** Average MAE (±SE) curves for age prediction are shown in blue, and their confidence intervals with each time window size are shown in green. **(C)** The height of the bar in the figure indicates the upper border of the permuted *P*-value for correlation coefficients of the brain score and age with each time window size. **(D)** The figure reflects the stability of the region bootstrap ratios (1/2 the sum of the connection bootstrap ratios to and from the regions) with changing time window size, suggesting the stability of the spatial patterns of maturation-dependent connections. Due to the large number of regions, only the “predictive regions” were selected and shown. Positive values indicate regions with connections mainly showing increased dynamics with age, and negative values denote regions with connections showing decreased dynamics.

To measure the impact of the sliding window size on prediction performance, the correlation coefficient (**Figure 5A**) between brain scores and age, MAE of prediction (**Figure 5B**), and permuted *P*-value (**Figure 5C**) of the correlation coefficient were calculated for each window size (from 20 to 80 s). To evaluate the effects on spatial patterns of maturation-dependent FC, “region bootstrap ratios” (1/2 the sum of the bootstrap ratios of all the connections to and from that region) were calculated and then scaled to an interval from -1 to 1. The significant regions with the top 50% of absolute region bootstrap ratios are shown in **Figure 5D**, and all regions are shown in Supplementary Figure S5. We found that the absolute prediction errors varied slowly with window sizes from 30 to 64 s. However, errors increased with window sizes shorter than 30 s or longer than 64 s. Furthermore, the permuted P-value of the correlation coefficient significantly increased, and the region bootstrap ratios became unstable above the window size thresholds. These results demonstrated that the influence of window size on PLS results was minimal when windows between 30 and 64 s were selected.

### Reproducibility

Inter-subject variability and experimental parameters, such as the sampling period of the fMRI data, may have a potential impact on the robust of dynamic properties of FC. In addition to the currently reported results on all 183 subjects, we also selected the two separate subsets to verify the reliability of the results. The two subsets differ in repetition time so that the impact of the sampling period could be evaluated. The correlation coefficient (*R*) between the bootstrap ratios of predictive connections was used as the similarity measurement. We found that the two subsets have a high degree of similarity in age-dependent patterns of connectivity, which is also similar to that of all the subjects (*R* = 0.94, *P* < 0.001; refer to Supplementary Figure S3). The bootstrap ratios of part of the connections exhibit differences, however, and a majority of predictive regions and connections are maintained across analyses of the different groups and all the subjects. Furthermore, network bootstrap ratios matrices, reflecting the distribution of age-dependent connectivity in networks, are reproducible (refer to **Figure 4**). Both of the groups have a high prediction accuracy (for the first group, MAE = 4.7 years, SE = 0.3 years, and for the second group, MAE = 4.8 years, SE = 0.4 years).

## Discussion

In this study, we confirmed the presence of a strong correlation between spontaneous fluctuation of resting-state FC and maturation age. Connections with increasing or decreasing fluctuation variability with maturity were mainly distributed between the specific ICNs. Accurate prediction of individual brain maturity using a predictive model based on the temporal variability of these connections further demonstrated that maturation information was encoded in the spontaneous fluctuations of specific resting-state functional connections. Furthermore, the identified predictive connections with increasing dynamics were mainly located between the cerebellum and the occipital network and DMN, as well as within the cerebellum and DMN. In contrast, connections with decreasing dynamics were found within the cerebellum and between the cerebellum and other networks, including the CON and DMN. These results suggested there are significant developmental changes in dynamic functional interaction between the major brain networks, which may provide new information about the functional organization of typical developmental brains.

### Functional Connectivity Fluctuations Decode Individual Brain Maturity

We have demonstrated the feasibility of predicting individual brain maturity based on low-frequency fluctuation of specific FC. We described the use of a novel measure, termed ALFF-FC, which characterizes the temporal variability of spontaneous fluctuation in resting-state FC. This measure was previously used to measure the total power of spontaneous neural activity within a specific frequency range (Yang et al., 2007) and has been applied to the resting-state fMRI studies of health and disease (Han et al., 2011; Yu et al., 2014). Recent studies of dynamic FC have shown that resting-state FC is not completely static, and changes generally manifest as movements from one short-term state to another rather than as continuous shifts (Hutchison et al., 2013a). If the connections become more variable with age, overall FC strength may become lower; otherwise, it may show the opposite effect (Betzel et al., 2014). Thus, the amplitude of oscillation in FC would reflect the frequency of information exchange and functional interaction between anatomically distinct regions. For example, a connection with the weaker amplitude of low-frequency fluctuation may mean more stable communication between distinct regions. Consequently, a whole-brain ALFF-FC map would provide new important information about dynamical organization of resting-state brains. In fact, individual differences in FC variability have been suggested to correlate with the tendency to attend to pain (Kucyi et al., 2013). Moreover, the degree to which a subject’s mind wanders away from a sensory stimulus has also been related to the variability of dynamic FC (Kucyi and Davis, 2014).

Increasing evidence supports the idea that major changes in cognitive and emotional functions from childhood to adolescence are the result of important refinements in complex neural dynamics and a reflection of the organization of the human brain (Kelly et al., 2009; Stevens et al., 2009; Dosenbach et al., 2010). For example, investigations into the organizing principles of this development found a developmental tendency toward functional segregation, which occurs through the weakening of short-range functional connections and the strengthening of long-range functional connections (Fair et al., 2009; Dosenbach et al., 2010). Further studies have demonstrated that children have stronger subcortical-cortical and weaker cortico-cortical connectivity compared to young adults (Supekar et al., 2009). Our results extend these findings on the developmental changes of static FC in response to dynamic aspects by demonstrating that the temporal variability of certain specific functional connections is significantly linked with the functional maturity of the brain. Our finding that fluctuation amplitude in some specific functional connections changes with age likely reflects developmental trends of functional organization in distinct brain systems. In addition, our results provide new evidence for the recent suggestion that spontaneous FC fluctuation is not just “noise” but rather tracks meaningful neural phenomena (Hutchison et al., 2013a).

### Specific Brain Networks Exhibit Changed Connectivity Fluctuation with Age

Separately summing the feature weights for each network (**Figure 4**) revealed that the DMN has a great relative feature weight for predicting functional maturity. As a hub for distant connections and a core functional network, the DMN plays a vital role in fundamental functions, such as self-relevant internal information processing (Raichle and Snyder, 2007) and monitoring the external environment (Hahn et al., 2007). A previous multimodal imaging study (combining resting-state fMRI, voxel-based morphometry and diffusion tensor imaging-based tractography) demonstrated that the DMN significantly changes with brain development both in functional and structural connectivity (Supekar et al., 2010). Many resting-state and task-based MRI studies also have found developmental changes in the DMN (Dosenbach et al., 2010; Grady et al., 2010; Sambataro et al., 2010; Meier et al., 2012; Sato et al., 2014). For example, the coherence of spontaneous activity in the DMN strengthens with maturation according to FC analyses (Fair et al., 2008; Kelly et al., 2009). The development of both the DMN and CON, with integration between the posterior and anterior neuronal modules is obviously observed (Sato et al., 2014). Furthermore, atypical DMN connectivity in attention-deficit/hyperactivity disorder (ADHD) may be involved in the delay and disruption of maturation (Fair et al., 2010). Current evidence supports the presence of changes in the dynamic FC within the DMN and between the DMN and other networks with age, confirming the crucial role of the DMN in functional brain maturation.

Another large-scale association network exhibiting a strong predictive power for brain maturity is the CON. This network is responsible for various cognitive processes and is a key component of control-related brain networks (Sadaghiani et al., 2010). fMRI studies have found that the CON exhibited a sizable weight in the prediction of functional maturity (Dosenbach et al., 2010; Meier et al., 2012). In particular, the caudate nucleus and thalamus were significantly associated with to brain maturation. Developmental interactions of the thalamus and the cerebellum with age have been found during inhibitory control tasks (Rubia et al., 2007). Furthermore, the variability of voxels within the middle temporal gyrus was increased with brain development (Garrett et al., 2010). In particular, we found a decreased fluctuation with age in the connections between the CON and cerebellum, suggesting increasing stability of the interactions and communication between these networks during functional brain maturation.

It should be noted that certain dynamic connections associated with the cerebellum exhibited a relatively high predictive power regarding brain maturity. Past resting-state and task neuroimaging studies have suggested that the cerebellum plays an important role in functional brain maturation (Rubia et al., 2007; Dosenbach et al., 2010). Abnormal functional maturation of cerebellar regions could help explain the cause of certain neurodevelopmental disorders, such as autism (Challis et al., 2015). Anatomical studies have demonstrated that major portions of the cerebellum are connected to cerebral association regions. Additionally, these cerebellar regions are functionally dedicated to cerebral association networks, with the exceptions of the primary visual and auditory cortices (Buckner et al., 2011). Based on these findings, we suggested that these observed age-dependent connections with the cerebellum may represent the normal maturation patterns of the cerebro-cerebellar circuit (Hunyadi et al., 2015) and underlie the development of motor and cognitive function from childhood to adulthood.

### Inter-Network Rather than Within-Network Connectivity Dynamics Shows Strong Developmental Trends

With respect to age-dependent connections, we can see that the internetwork functional connections are more extensively represented than are the intra-network connections, except for that of the cerebellum (**Figure 4C**). The connections with the greatest predictive power are mainly distributed across the DMN, CON, and cerebellum (**Figures 4A,B**), suggesting the significant development of inter-network dynamic interactions in addition to the intra-network connection alterations reported previously in these areas. This result is consistent with findings of functional brain network development, which demonstrate the organization of multiple functional networks, mainly involving the CON, fronto-parietal network, DMN, the cerebellar network, shifting from a local anatomical emphasis in children to a more “distributed” architecture in young adult during development (Fair et al., 2009).

The findings of inter-network interaction changes presented here are also consistent with the recent suggestion that perceptual and cognitive development involve the simultaneous segregation and integration of processing streams (Johnson, 2001; Luna and Sweeney, 2004; Bunge and Wright, 2007; Fair et al., 2007). We observed that obvious decreases in dynamic connection variability occur between the DMN and CON, as well as between the CON and cerebellum (**Figure 4A**). Given that connections that are less variable with age converge toward higher overall FC strength, these results are consistent with earlier findings that static FC tends to increase with age between higher-order cognitive systems. For example, increased connectivity with age between the DMN and the control network as well as the ventral attention network has been reported (Geerligs et al., 2015). In contrast, the fluctuation of connections between the occipital cortex and the cerebellum and between the DMN and the cerebellum exhibits a significant, linear increase during development (**Figure 4B**). Age-related differences in the frequency of state expression may provide a possible explanation of the different developmental trends in between-network FC (Hutchison and Morton, 2015). For example, older participants express several connectivity states more frequently than younger participants, while the opposite relationship is observed for other connectivity states. These specific patterns of dynamic interactions across functional networks carry a large volume of developmental information from the various processing streams that underlie the brain’s cognitive and emotional functions.

An alternative interpretation of these results is based on the structural connectivity changes that occur with age, as the temporal stability of FC has been empirically demonstrated to be dependent on structural topology (Shen et al., 2015). One of the significant trends in anatomical changes with age is the reduction in the number of edges for the transmission of neural signals, resulting in an increase in multi-step paths of functional communication to support the high FC (Betzel et al., 2014). This likely leads to more variable functional connections between specific regions, especially in higher-order cognition-related cortices. For instance, EEG and MEG studies of infants and children up to 15 years-old have confirmed that brain signal variability increases with age and that greater variability is correlated with higher cognitive performance (McIntosh et al., 2008; Mišić et al., 2010). In particular, this increase in the entropy of brain activity could be attributed to the widespread exchange of information between distal brain regions, rather than an increase in local dynamics (Vakorin et al., 2011).

### Control Analysis, Limitations, and Directions for Future Research

It has been shown that head movement artifacts could bias measurements of FC by decreasing long-distance correlations and increasing short-distance correlations (Satterthwaite et al., 2012; Van Dijk et al., 2012), thereby having a potential impact on developmental studies that evaluate FC (Dosenbach et al., 2010). Head movement confounding is of particular importance during maturation studies because motion is strongly related to subjective age in children (Seto et al., 2001;Van Dijk et al., 2012). On the one hand, we hypothesized in the present study that low-frequency fluctuations in FC may contain head movement artifacts, and we investigated the present results after regressing out the mean head movement time course from the time series of dynamic FC. On the other hand, given that head motion has a neurobiological basis (Zeng et al., 2014), which is likely dependent on age, a direct regression of averaged head motion signals on the ALFF-FC values for individual subjects was also performed. In both cases, our results revealed few changes, even after adjusting for movement, suggesting that head motion had a limited impact on the fundamental patterns of developmental changes in the dynamics of resting-state FC during maturation (Supplementary Figure S5). In fact, no connections exhibited a significant correlation between averaged head motion and ALFF-FC (FDR-corrected *P* < 0.05). In order to further demonstrate that the results are not motion-related, we repeated the analysis on a subset of subjects where age is uncorrelated with head motion, found that the correlation between the brain scores and ages remained (Supplementary Figure S6). Together, these results of the control analyses demonstrated that head motion has a limited impact on the fundamental patterns of developmental changes in the dynamics of resting-state FC during maturation. This conclusion is consistent with those of other developmental studies using this dataset that have already quantified the micro movements of the subjects (Betzel et al., 2014).

We also investigated the stability of the identified predictive connections and corresponding prediction performance with varying sliding window sizes. Interestingly, we found that window sizes of 30-60 s achieved the best prediction performance (**Figures 5A–C**). More importantly, the identified “predictive connections” that made the greatest contribution to prediction converged within this range of windows lengths (**Figure 5D**). This result suggests that durations of 30–60 s are short enough to capture interesting transient events that decode the functional maturity of brains. However, **Figures 5B,C** demonstrate a significant decrease in prediction accuracy when using longer (>60 s) windows. At the same time, the predictive connections also become unstable (**Figure 5D**). These results are consistent with a previous finding indicating that variability across longer windows does not adequately capture the dynamics of spontaneous cognition (Shirer et al., 2012; Kucyi and Davis, 2014). Additionally, an excessively small window size also results in poor prediction performance (**Figures 5B,C**) due to the reduced number of time points, which results in a decreased SNR (Hutchison et al., 2013a).

We found that the averaged SNR maps of the young and adult groups were similar (see Supplementary Figure S7), and the *t-*test used to compare the SNR maps of the groups did not reveal any significant regions (*P* < 0.05, FDR corrected). Moreover, compared to the results without SNR regression, SNR regression had little effect on the performance of the prediction between age and brain scores, *R* = 0.701, *P* < 0.0001). Overall, the results indicated that the SNR maps of the subjects did not exhibit significant differences with age and that the largest differences in the cerebellum and visual cortex were likely not due to excessive artifacts.

In the present study, we attempted to decrease the impact of these nuisances on the signals of interest as far as possible, by filtering the original BOLD signals using a more narrow frequency band from 1/*w* (*w* is a windows length) to 0.08 Hz (Glerean et al., 2012), low-pass filtering correlation coefficients timecourses with the cut-off frequency 1/*w* (Leonardi and Van De Ville, 2015) and regressing CSF signals from the BOLD signals. However, one should also consider that fluctuations of dynamic FC could partly be driven by time-varying noise (e.g., head motion and variable respiratory and cardiac rhythms, which correlate with age during maturation), despite our attempts to minimize the influences of these confounding factors. The observed distinctions between identified regions associated with the predictive connections and the previously reported heartbeat-dependent or respiration-related regions offer some confirmation that the observed effects are not solely due to non-specific physiological alteration. Future research should further validate to what extent these changes of dynamic FC reflect developmental trends of underlying neurophysiological signals, using concurrent measurements such as EEG and non-neurophysiological signals.

Some of the results presented here should be considered in the context of several experimental and methodological limitations. First, respiration and heart-rate signals were not provided in the NKI sample. Although we attempted to weaken the impact of these physiological nuisance factors by regressing out the WM and CSF signals, the use of more sophisticated tools for the removal of nuisance factors and artifacts, such as ICA+ Fix (Smith et al., 2013), would be expected to further remove the nuisance factors. Second, other measures of variability, such as the autocorrelation coefficients of FC time series (Shen et al., 2015), have been applied to estimate the temporal stability of FC. The reliability of the results using different measures of temporal variability should be tested in future studies. Finally, a limitation of resting-state FC MRI in general is the restricted frequency distribution when measuring correlations, which is typically below 0.1 Hz. There is also the possibility of undetected developmental changes that manifest as dynamic correlations in other frequency distributions. Including other imaging and psychometric techniques, such as simultaneous EEG-fMRI, will likely help address these considerations. Specifically, future work that investigates a direct relationship between behavior and the variability of dynamic FC is needed to deepen our understanding of the developmental trajectory of dynamic FC.

## Conclusion

Using a sliding window approach based on resting-state fMRI data, we provided the first evidence of developmental changes in the amplitude of low-frequency spontaneous fluctuations in resting-state FC. Various age-dependent changes have been found within and between several specific ICNs of human brains, suggesting a typical maturation pattern of dynamic interaction and communication across the major brain networks. This developmental tendency in the temporal properties of FC deepens our understanding of functional network dynamics of typical brain development and will help expand research into the relationship between the variability of low-frequency oscillations in resting-state FC and the development of cognitive and perceptual abilities from childhood to adulthood.

## Conflict of Interest Statement

The authors declare that the research was conducted in the absence of any commercial or financial relationships that could be construed as a potential conflict of interest.

## Footnotes

1 http://fcon.1000.projects.nitrc.org/indi/pro/nki.html

2 http://www.fil.ion.ucl.ac.uk/spm

3 www.fmrib.ox.ac.uk/fsl

4 http://www.ansir.wfubmc.edu
