# Supplementary material for: Predicting individual brain maturity using dynamic functional connectivity
Source: Front Hum Neurosci. 2015 Jul 16;9:418. doi: 10.3389/fnhum.2015.00418 (PMC4503925; doi:10.3389/fnhum.2015.00418)
Supplement: Supplementary file 1 [file Data_Dheet_1.DOC]

***Supplementary Material***

**Predicting individual brain maturity using dynamic functional connectivity**

**Jian Qin^1^, Shan-Guang Chen^2^, Dewen Hu^1^, Ling-Li Zeng^1^, Yi-Ming Fan^1^, Xiao-Ping Chen^2*^, Hui Shen^1*^**

^1^College of Mechatronics and Automation, National University of Defense Technology, Changsha, Hunan 410073, China

^2^National Key Laboratory of Human Factors Engineering, China Astronaut Research and Training Center, Beijing 100094, China

***Correspondence:**

Xiao-Ping Chen, National Key Laboratory of Human Factors Engineering, China Astronaut Research and Training Center, Haidian District, Beijing 100094, China

E-mail: xpchen2009@163.com

Hui Shen, College of Mechatronics and Automation, National University of Defense Technology, Changsha 410073, China.

E-mail: [shenhui_nudt@126.com](mailto:shenhui_nudt@126.com)

1. **Supplementary Figures and Tables**
   1. **Supplementary Figures**

**
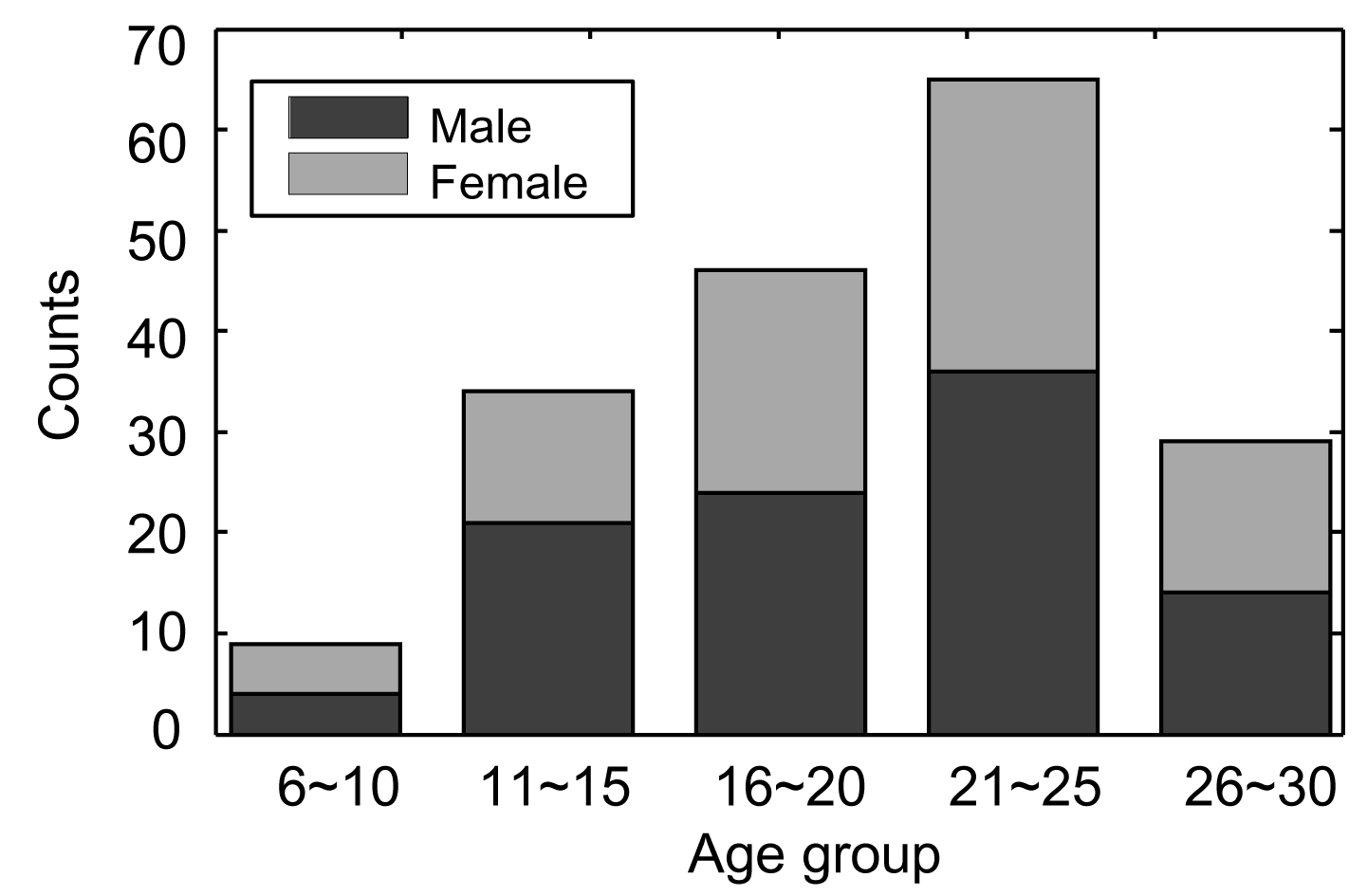
**

**Figure S1**. Distribution of the subjects according to age and sex.


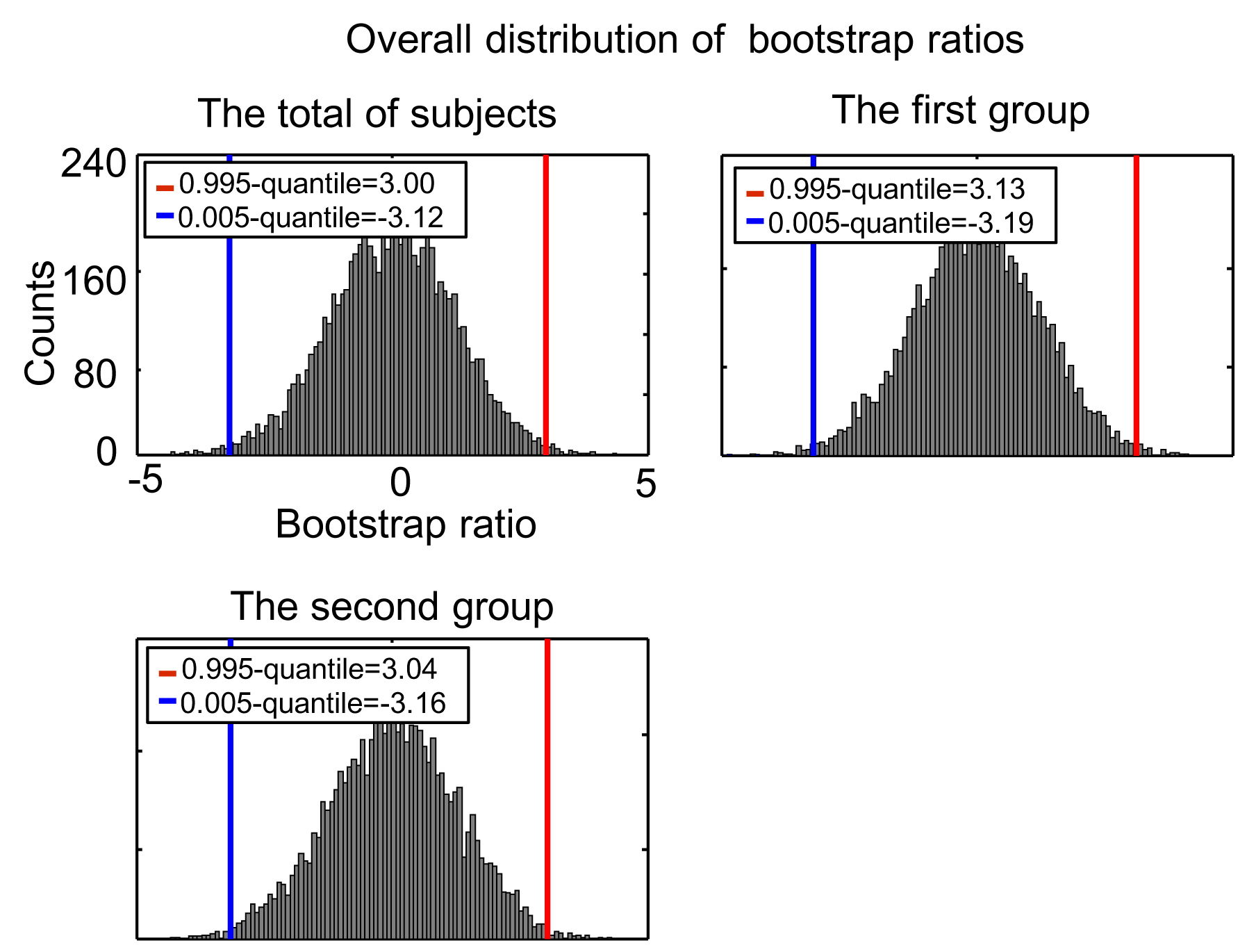


**Figure S2.** Overall distribution of the bootstrap ratios.


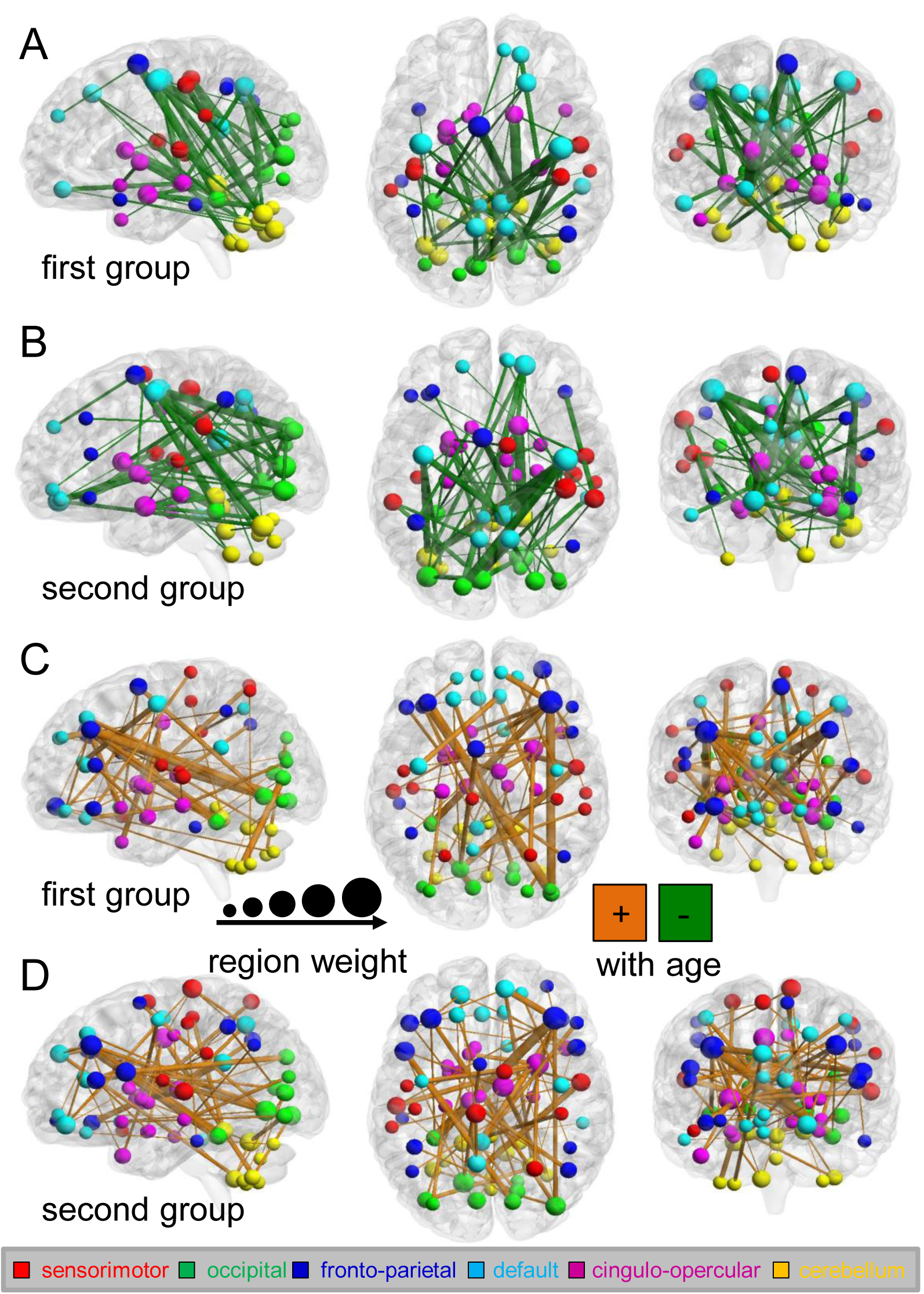


**Figure S3.** Age-dependent connections and regions in the dynamics of functional connectivity from the analysis on the first and second groups. Only connections with bootstrap ratios over 3.0 were selected and considered predictive connections. (A) and (B) Functional connections that decrease with maturity during the PLS analysis (shown in green) and age-dependent ROIs are displayed on the surface of the brain. Connection lines are scaled according to the bootstrap ratios. The color and scale of ROIs represent the functional networks and region weights (1/2 the sum of connection bootstrap ratios to and from that region), respectively. In contrast, (C) and (D) functional connections that increase with maturity are displayed in orange.


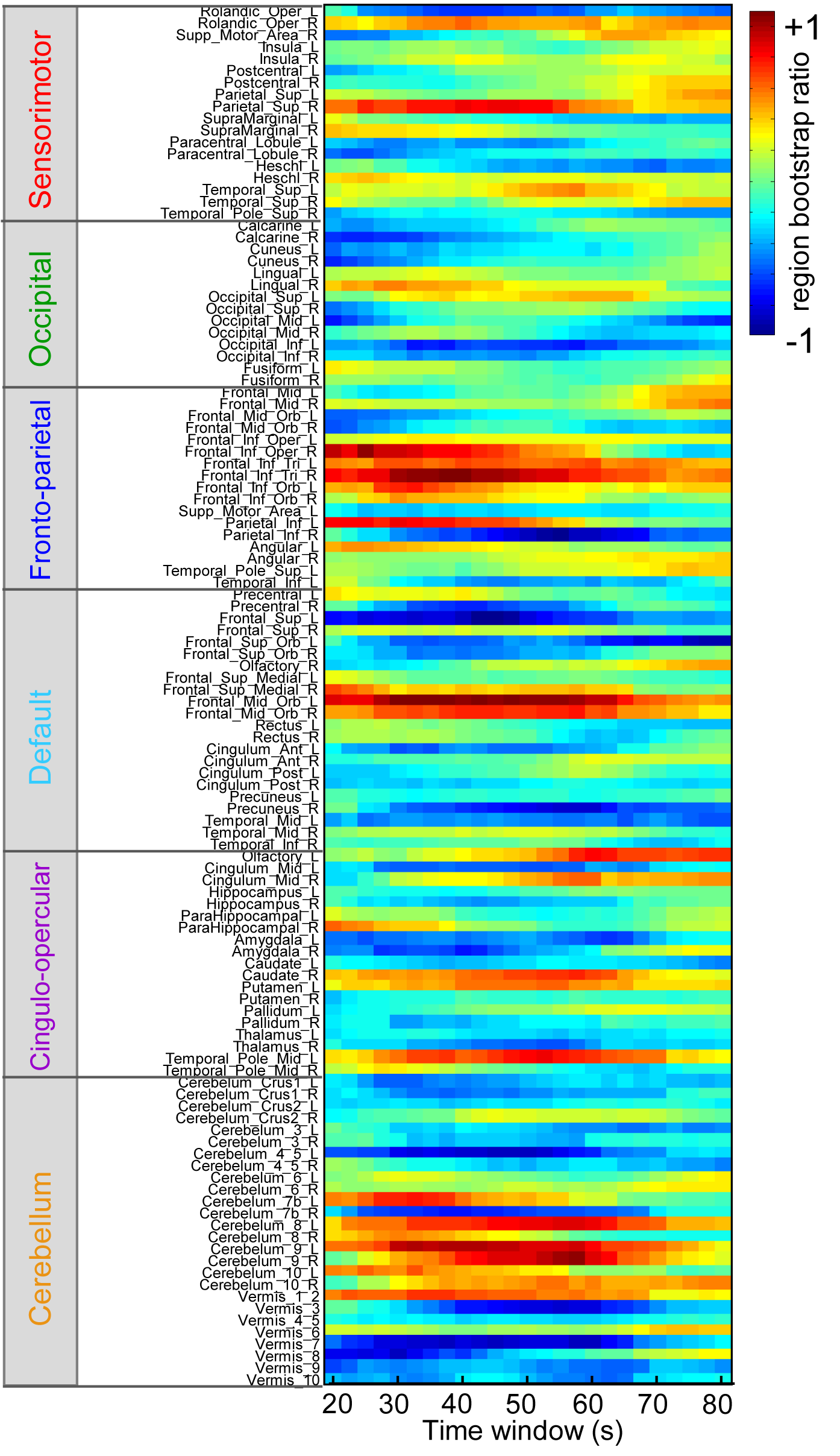


**Figure S4.** Effect of the slice window size on the PLS results. The figure reflects the stability of the region bootstrap ratio (1/2 the sum of connection bootstrap ratios to and from the regions) with changing time window sizes, suggesting the stability of the spatial pattern of age-dependent connections. Positive values indicate regions with connections mainly showing increased dynamics with age and negative values denote regions with connections showing decreased dynamics.

**
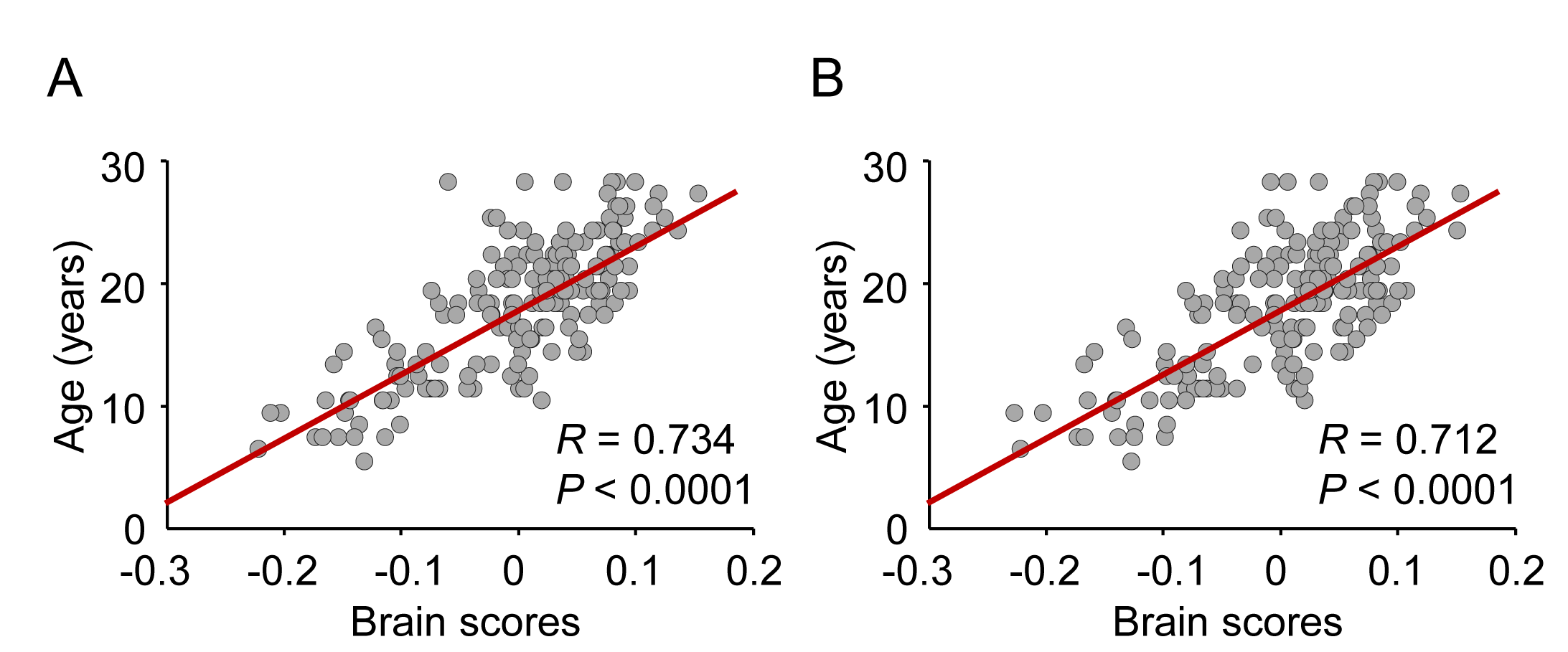
**

**Figure S5.** Influence of head motion on prediction performance. (A) The relationship between age and brain scores after removing head motion across sliced windows. (B) The relationship between age and brain scores after removing the mean head motion across subjects.

**
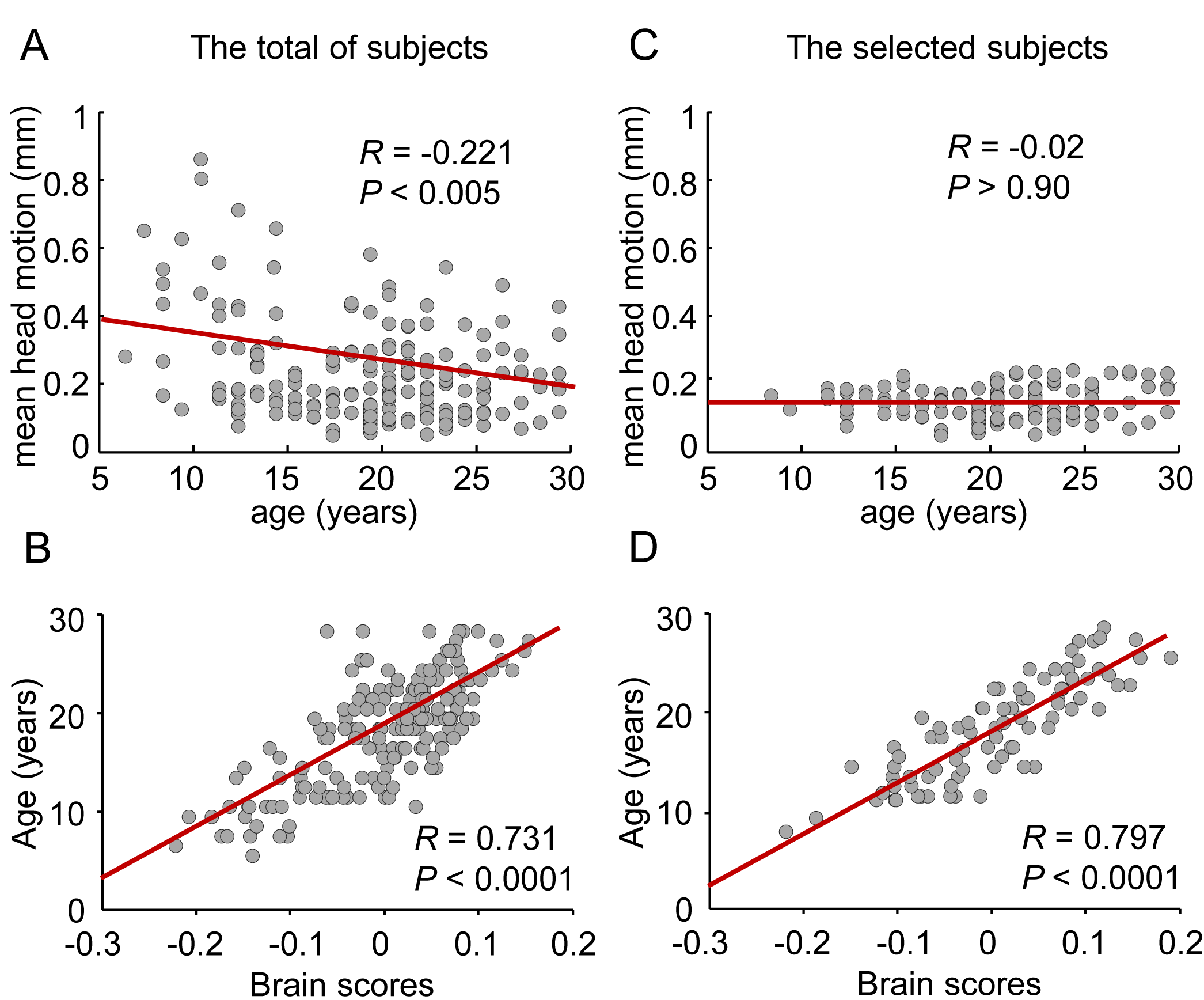
**

**Figure S6.** The relationship between mean head motion (defined in Materials and Methods) and ages, and motion influences. (A) The negative correlation (*R* = -0.221, *P* < 0.005) between mean head motion and ages in the total of subjects. (B) The significant relationship between brain scores and ages of the total of subjects. (C) A selected subset of subjects whose motion is uncorrelated with ages (*P* > 0.90). (D) The significant correlation between brain scores and ages was observed, even if the level of head motion in the selected subjects has been controlled.


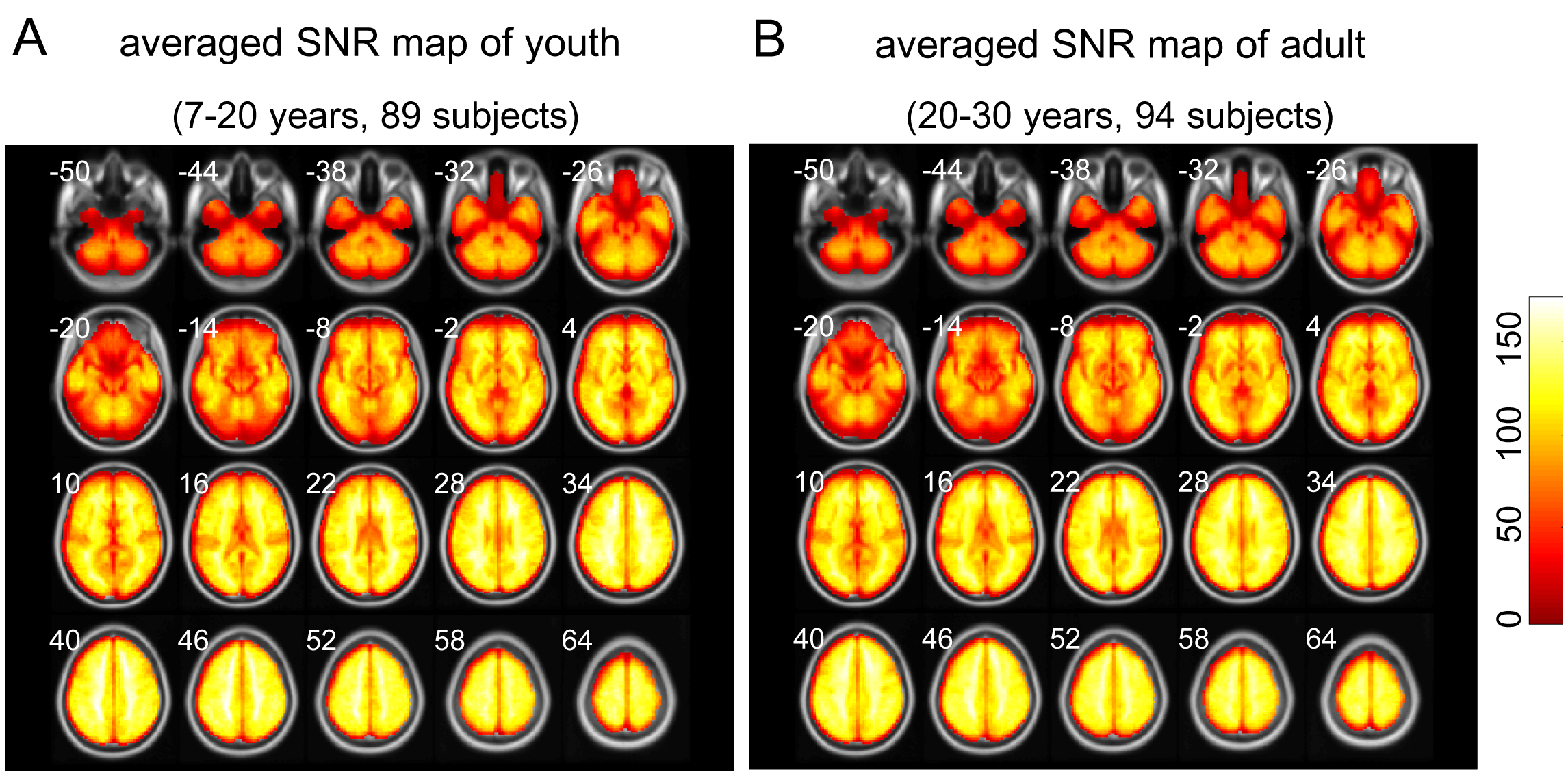


**Figure S7.** The averaged signal-to-noise ratio (SNR) maps of the youth and adult groups. (A) and (B) show the averaged SNR maps of the youth and adult groups, respectively. The SNR map was calculated for each voxel by averaging the signal intensity across the whole run and dividing it by the standard deviation over time.

- 1. **Supplementary Tables**

**Table S1.** The cut-off frequency and data points for given window sizes.

| window size (*s*) | 20 | 25 | 30 | 35 | 40 | 45 | 50 | 55 | 60 | 65 | 70 | 75 | 80 |
| --- | --- | --- | --- | --- | --- | --- | --- | --- | --- | --- | --- | --- | --- |
| cutoff frequency (Hz) | .050 | .040 | .033 | .029 | .025 | .022 | .020 | .018 | .017 | .015 | .014 | .013 | .013 |
| data points  (TR = 2.5 s) | 247 | 245 | 243 | 241 | 239 | 237 | 235 | 233 | 231 | 229 | 227 | 225 | 223 |
| data points  (TR = 0.645 s) | 863 | 856 | 848 | 840 | 832 | 825 | 817 | 809 | 801 | 794 | 786 | 778 | 770 |

Given the window size (*w*), the cutoff frequency is 1/*w*. The total data points of different data types are 250 and 900, respectively, but the first five scans both were discarded in the data preprocessing.

**Table S2**. Summed bootstrap ratios and labels of brain regions.

| **MNI coordinates** | | | **Network** | **Region label** | **Summed  bootstrap ratios** |
| --- | --- | --- | --- | --- | --- |
| **x** | **y** | **z** |
| 9 | 0 | 62 | sensorimotor | Supp_Motor_Area_R | 8.9 |
| -53 | -21 | 7 | sensorimotor | Temporal_Sup_L | 6.7 |
| 53 | -6 | 15 | sensorimotor | Rolandic_Oper_R | 3.6 |
| 41 | -25 | 53 | sensorimotor | Postcentral_R | 3.4 |
| -8 | -25 | 70 | sensorimotor | Paracentral_Lobule_L | 3.1 |
| 46 | -17 | 10 | sensorimotor | Heschl_R | 1.7 |
| 58 | -32 | 34 | sensorimotor | SupraMarginal_R | 1.7 |
| 26 | -59 | 62 | sensorimotor | Parietal_Sup_R | 1.6 |
| -47 | -8 | 14 | sensorimotor | Rolandic_Oper_L | 1.6 |
| 39 | 6 | 2 | sensorimotor | Insula_R | 1.6 |
| 58 | -22 | 7 | sensorimotor | Temporal_Sup_R | 1.5 |
| -7 | -79 | 6 | occipital | Calcarine_L | 9.5 |
| 16 | -67 | -4 | occipital | Lingual_R | 8.9 |
| -15 | -68 | -5 | occipital | Lingual_L | 5.4 |
| 14 | -79 | 28 | occipital | Cuneus_R | 5.0 |
| 24 | -81 | 31 | occipital | Occipital_Sup_R | 3.8 |
| 16 | -73 | 9 | occipital | Calcarine_R | 3.5 |
| -31 | -40 | -20 | occipital | Fusiform_L | 3.4 |
| 38 | -82 | -8 | occipital | Occipital_Inf_R | 3.2 |
| -17 | -84 | 28 | occipital | Occipital_Sup_L | 3.1 |
| 34 | -39 | -20 | occipital | Fusiform_R | 1.7 |
| 38 | 33 | 34 | fronto-parietal | Frontal_Mid_R | 12.5 |
| 46 | -46 | 50 | fronto-parietal | Parietal_Inf_R | 6.5 |
| -48 | 13 | 19 | fronto-parietal | Frontal_Inf_Oper_L | 4.8 |
| -33 | 33 | 35 | fronto-parietal | Frontal_Mid_L | 3.4 |
| 33 | 53 | -11 | fronto-parietal | Frontal_Mid_Orb_R | 3.1 |
| 46 | -60 | 39 | fronto-parietal | Angular_R | 1.6 |
| -46 | 30 | 14 | fronto-parietal | Frontal_Inf_Tri_L | 1.6 |
| -44 | -61 | 36 | fronto-parietal | Angular_L | 1.6 |
| 50 | 30 | 14 | fronto-parietal | Frontal_Inf_Tri_R | 1.5 |
| -5 | 5 | 61 | fronto-parietal | Supp_Motor_Area_L | 1.5 |
| -5 | 49 | 31 | default | Frontal_Sup_Medial_L | 6.8 |
| 9 | 51 | 30 | default | Frontal_Sup_Medial_R | 5.8 |
| 10 | 16 | -11 | default | Olfactory_R | 5.6 |
| 54 | -31 | -22 | default | Temporal_Inf_R | 5.5 |
| -5 | -43 | 25 | default | Cingulum_Post_L | 3.7 |
| -5 | 54 | -7 | default | Frontal_Mid_Orb_L | 3.1 |
| **MNI coordinates** | | | **Network** | **Region label** | **Summed  bootstrap ratios** |
| **x** | **y** | **z** |
| 10 | -56 | 44 | default | Precuneus_R | 1.7 |
| -18 | 35 | 42 | default | Frontal_Sup_L | 1.7 |
| -17 | 47 | -13 | default | Frontal_Sup_Orb_L | 1.7 |
| 8 | 52 | -7 | default | Frontal_Mid_Orb_R | 1.7 |
| -7 | -56 | 48 | default | Precuneus_L | 1.6 |
| 8 | 36 | -18 | default | Rectus_R | 1.5 |
| -8 | 15 | -11 | cingulo-opercular | Olfactory_L | 6.4 |
| 28 | 5 | 2 | cingulo-opercular | Putamen_R | 5.0 |
| 8 | -9 | 40 | cingulo-opercular | Cingulum_Mid_R | 4.9 |
| -24 | 4 | 2 | cingulo-opercular | Putamen_L | 4.8 |
| -36 | 15 | -34 | cingulo-opercular | Temporal_Pole_Mid_L | 3.3 |
| 27 | 1 | -18 | cingulo-opercular | Amygdala_R | 3.2 |
| -11 | 11 | 9 | cingulo-opercular | Caudate_L | 3.1 |
| 44 | 15 | -32 | cingulo-opercular | Temporal_Pole_Mid_R | 1.7 |
| -11 | -18 | 8 | cingulo-opercular | Thalamus_L | 1.6 |
| -18 | 0 | 0 | cingulo-opercular | Pallidum_L | 1.6 |
| -26 | -55 | -48 | cerebellum | Cerebelum_8_L | 8.8 |
| 9 | -50 | -46 | cerebellum | Cerebelum_9_R | 5.5 |
| -11 | -49 | -46 | cerebellum | Cerebelum_9_L | 3.6 |
| 0 | -46 | -32 | cerebellum | Vermis_10 | 3.2 |
| 33 | -63 | -48 | cerebellum | Cerebelum_7b_R | 3.1 |
| 1 | -39 | -20 | cerebellum | Vermis_1_2 | 1.8 |
| 17 | -43 | -18 | cerebellum | Cerebelum_4_5_R | 1.7 |
| 1 | -67 | -15 | cerebellum | Vermis_6 | 1.7 |
| 25 | -56 | -49 | cerebellum | Cerebelum_8_R | 1.6 |
| -23 | -34 | -42 | cerebellum | Cerebelum_10_L | 1.6 |
| 1 | -72 | -25 | cerebellum | Vermis_7 | 1.6 |
| -32 | -60 | -45 | cerebellum | Cerebelum_7b_L | 1.6 |
| -56 | -34 | 30 | sensorimotor | SupraMarginal_L | -8.9 |
| 58 | -22 | 7 | sensorimotor | Temporal_Sup_R | -3.8 |
| -42 | -23 | 49 | sensorimotor | Postcentral_L | -3.5 |
| 41 | -25 | 53 | sensorimotor | Postcentral_R | -3.3 |
| -42 | -19 | 10 | sensorimotor | Heschl_L | -3.2 |
| 7 | -32 | 68 | sensorimotor | Paracentral_Lobule_R | -3.2 |
| -17 | -84 | 28 | occipital | Occipital_Sup_L | -10.6 |
| 16 | -67 | -4 | occipital | Lingual_R | -10.6 |
| -6 | -80 | 27 | occipital | Cuneus_L | -10.5 |
| -7 | -79 | 6 | occipital | Calcarine_L | -5.5 |
| **MNI coordinates** | | | **Network** | **Region label** | **Summed  bootstrap ratios** |
| **x** | **y** | **z** |
| 14 | -79 | 28 | occipital | Cuneus_R | -3.2 |
| 24 | -81 | 31 | occipital | Occipital_Sup_R | -3.2 |
| -31 | -40 | -20 | occipital | Fusiform_L | -1.9 |
| 16 | -73 | 9 | occipital | Calcarine_R | -1.8 |
| 38 | -82 | -8 | occipital | Occipital_Inf_R | -1.7 |
| -36 | -78 | -8 | occipital | Occipital_Inf_L | -1.7 |
| 46 | -46 | 50 | fronto-parietal | Parietal_Inf_R | -12.4 |
| -5 | 5 | 61 | fronto-parietal | Supp_Motor_Area_L | -9.1 |
| -43 | -46 | 47 | fronto-parietal | Parietal_Inf_L | -5.2 |
| -33 | 33 | 35 | fronto-parietal | Frontal_Mid_L | -4.8 |
| 46 | -60 | 39 | fronto-parietal | Angular_R | -3.2 |
| -31 | 50 | -10 | fronto-parietal | Frontal_Mid_Orb_L | -1.5 |
| 41 | -8 | 52 | default | Precentral_R | -12.0 |
| 10 | -56 | 44 | default | Precuneus_R | -7.8 |
| -39 | -6 | 51 | default | Precentral_L | -7.4 |
| 7 | -42 | 22 | default | Cingulum_Post_R | -6.4 |
| -7 | -56 | 48 | default | Precuneus_L | -4.7 |
| -5 | 37 | -18 | default | Rectus_L | -1.9 |
| 9 | 51 | 30 | default | Frontal_Sup_Medial_R | -1.7 |
| 18 | 48 | -14 | default | Frontal_Sup_Orb_R | -1.7 |
| 8 | 52 | -7 | default | Frontal_Mid_Orb_R | -1.6 |
| -5 | -43 | 25 | default | Cingulum_Post_L | -1.6 |
| 8 | 36 | -18 | default | Rectus_R | -1.6 |
| 15 | 12 | 9 | cingulo-opercular | Caudate_R | -5.0 |
| -25 | -21 | -10 | cingulo-opercular | Hippocampus_L | -3.8 |
| -24 | 4 | 2 | cingulo-opercular | Putamen_L | -2.3 |
| 21 | 0 | 0 | cingulo-opercular | Pallidum_R | -1.8 |
| -23 | -1 | -17 | cingulo-opercular | Amygdala_L | -1.7 |
| 28 | 5 | 2 | cingulo-opercular | Putamen_R | -1.7 |
| -11 | -18 | 8 | cingulo-opercular | Thalamus_L | -1.7 |
| -21 | -16 | -21 | cingulo-opercular | ParaHippocampal_L | -1.7 |
| 8 | -9 | 40 | cingulo-opercular | Cingulum_Mid_R | -1.7 |
| -11 | 11 | 9 | cingulo-opercular | Caudate_L | -1.6 |
| 44 | 15 | -32 | cingulo-opercular | Temporal_Pole_Mid_R | -1.5 |
| -36 | 15 | -34 | cingulo-opercular | Temporal_Pole_Mid_L | -1.5 |
| 32 | -69 | -40 | cerebellum | Cerebelum_Crus2_R | -7.6 |
| 37 | -67 | -30 | cerebellum | Cerebelum_Crus1_R | -7.1 |
| -29 | -73 | -38 | cerebellum | Cerebelum_Crus2_L | -6.5 |
| **MNI coordinates** | | | **Network** | **Region label** | **Summed  bootstrap ratios** |
| **x** | **y** | **z** |
| 25 | -58 | -24 | cerebellum | Cerebelum_6_R | -6.2 |
| -36 | -67 | -29 | cerebellum | Cerebelum_Crus1_L | -4.7 |
| 17 | -43 | -18 | cerebellum | Cerebelum_4_5_R | -4.0 |
| -26 | -55 | -48 | cerebellum | Cerebelum_8_L | -3.4 |
| 33 | -63 | -48 | cerebellum | Cerebelum_7b_R | -3.3 |
| -23 | -34 | -42 | cerebellum | Cerebelum_10_L | -3.1 |
| 12 | -34 | -19 | cerebellum | Cerebelum_3_R | -1.8 |
| -9 | -37 | -19 | cerebellum | Cerebelum_3_L | -1.7 |
| 26 | -34 | -41 | cerebellum | Cerebelum_10_R | -1.7 |
| -15 | -43 | -17 | cerebellum | Cerebelum_4_5_L | -1.7 |
| 9 | -50 | -46 | cerebellum | Cerebelum_9_R | -1.6 |
| 1 | -64 | -34 | cerebellum | Vermis_8 | -1.6 |
| 1 | -52 | -6 | cerebellum | Vermis_4_5 | -1.5 |
| -11 | -49 | -46 | cerebellum | Cerebelum_9_L | -1.5 |
| 0 | -46 | -32 | cerebellum | Vermis_10 | -1.5 |

**Table S3**. Bootstrap ratio and label of each remarkable connection.

| **Region label A** | **Network A** | **Region label B** | **Network B** | **Bootstrap ratio** |
| --- | --- | --- | --- | --- |
| Postcentral_R | sensorimotor | Supp_Motor_Area_R | sensorimotor | 3.5 |
| Temporal_Sup_L | sensorimotor | Cuneus_R | occipital | 3.3 |
| Temporal_Sup_L | sensorimotor | Frontal_Mid_R | fronto-parietal | 3.5 |
| Temporal_Sup_R | sensorimotor | Frontal_Mid_Orb_R | fronto-parietal | 3.0 |
| Temporal_Sup_L | sensorimotor | Parietal_Inf_R | fronto-parietal | 3.5 |
| Paracentral_Lobule_L | sensorimotor | Frontal_Mid_Orb_L | default | 3.2 |
| Postcentral_R | sensorimotor | Olfactory_L | cingulo-opercular | 3.3 |
| Heschl_R | sensorimotor | Putamen_L | cingulo-opercular | 3.5 |
| Temporal_Sup_L | sensorimotor | Putamen_R | cingulo-opercular | 3.3 |
| Calcarine_L | occipital | Rolandic_Oper_L | sensorimotor | 3.2 |
| Lingual_L | occipital | Rolandic_Oper_R | sensorimotor | 3.4 |
| Lingual_L | occipital | Supp_Motor_Area_R | sensorimotor | 3.2 |
| Occipital_Sup_R | occipital | Supp_Motor_Area_R | sensorimotor | 3.3 |
| Lingual_R | occipital | Insula_R | sensorimotor | 3.2 |
| Occipital_Inf_R | occipital | Frontal_Mid_L | fronto-parietal | 3.2 |
| Occipital_Inf_R | occipital | Frontal_Mid_R | fronto-parietal | 3.3 |
| Fusiform_L | occipital | Frontal_Mid_R | fronto-parietal | 3.6 |
| Calcarine_R | occipital | Frontal_Inf_Oper_L | fronto-parietal | 3.0 |
| Cuneus_R | occipital | Frontal_Inf_Oper_L | fronto-parietal | 3.5 |
| Lingual_R | occipital | Frontal_Sup_L | default | 3.3 |
| Calcarine_R | occipital | Frontal_Sup_Medial_L | default | 3.8 |
| Occipital_Sup_L | occipital | Frontal_Sup_Medial_L | default | 3.0 |
| Occipital_Sup_R | occipital | Frontal_Sup_Medial_L | default | 3.5 |
| Cuneus_R | occipital | Olfactory_L | cingulo-opercular | 3.2 |
| Lingual_L | occipital | Cingulum_Mid_R | cingulo-opercular | 3.0 |
| Parietal_Inf_R | fronto-parietal | Fusiform_R | occipital | 3.3 |
| Parietal_Inf_R | fronto-parietal | Frontal_Inf_Oper_L | fronto-parietal | 3.0 |
| Parietal_Inf_R | fronto-parietal | Cingulum_Mid_R | cingulo-opercular | 3.3 |
| Rectus_R | default | Supp_Motor_Area_R | sensorimotor | 3.0 |
| Precuneus_R | default | Supp_Motor_Area_R | sensorimotor | 3.5 |
| Frontal_Sup_Medial_R | default | Frontal_Mid_L | fronto-parietal | 3.6 |
| Olfactory_R | default | Frontal_Mid_R | fronto-parietal | 3.8 |
| Frontal_Sup_Medial_R | default | Frontal_Sup_L | default | 4.2 |
| Cingulum_Post_L | default | Precuneus_R | default | 3.0 |
| Precuneus_L | default | Frontal_Sup_Medial_R | default | 3.2 |
| Frontal_Mid_Orb_R | default | Frontal_Sup_Orb_L | default | 3.3 |
| Frontal_Sup_Medial_R | default | Olfactory_R | default | 3.8 |
| Temporal_Inf_R | default | Putamen_L | cingulo-opercular | 3.2 |
| **Region label A** | **Network A** | **Region label B** | **Network B** | **Bootstrap ratio** |
| Temporal_Inf_R | default | Putamen_R | cingulo-opercular | 3.5 |
| Amygdala_R | cingulo-opercular | Rolandic_Oper_R | sensorimotor | 3.2 |
| Putamen_R | cingulo-opercular | Parietal_Sup_R | sensorimotor | 3.2 |
| Caudate_L | cingulo-opercular | Paracentral_Lobule_L | sensorimotor | 3.0 |
| Putamen_L | cingulo-opercular | Fusiform_L | occipital | 3.2 |
| Olfactory_L | cingulo-opercular | Frontal_Mid_R | fronto-parietal | 3.2 |
| Temporal_Pole_Mid_R | cingulo-opercular | Frontal_Mid_R | fronto-parietal | 3.2 |
| Temporal_Pole_Mid_L | cingulo-opercular | Frontal_Mid_Orb_R | fronto-parietal | 3.1 |
| Caudate_L | cingulo-opercular | Frontal_Inf_Tri_L | fronto-parietal | 3.0 |
| Amygdala_R | cingulo-opercular | Supp_Motor_Area_L | fronto-parietal | 3.0 |
| Thalamus_L | cingulo-opercular | Putamen_R | cingulo-opercular | 3.0 |
| Pallidum_L | cingulo-opercular | Olfactory_L | cingulo-opercular | 3.2 |
| Temporal_Pole_Mid_L | cingulo-opercular | Cingulum_Mid_R | cingulo-opercular | 3.5 |
| Vermis_6 | cerebellum | SupraMarginal_R | sensorimotor | 3.3 |
| Cerebelum_4_5_R | cerebellum | Calcarine_L | occipital | 3.3 |
| Cerebelum_8_L | cerebellum | Calcarine_L | occipital | 4.8 |
| Cerebelum_9_L | cerebellum | Calcarine_L | occipital | 3.6 |
| Cerebelum_9_R | cerebellum | Calcarine_L | occipital | 4.1 |
| Cerebelum_8_L | cerebellum | Lingual_R | occipital | 3.9 |
| Cerebelum_9_L | cerebellum | Lingual_R | occipital | 3.6 |
| Cerebelum_9_R | cerebellum | Lingual_R | occipital | 3.8 |
| Cerebelum_9_R | cerebellum | Occipital_Sup_L | occipital | 3.2 |
| Vermis_1_2 | cerebellum | Olfactory_R | default | 3.8 |
| Cerebelum_7b_L | cerebellum | Frontal_Sup_Medial_L | default | 3.4 |
| Cerebelum_7b_R | cerebellum | Frontal_Mid_Orb_L | default | 3.2 |
| Cerebelum_8_L | cerebellum | Cingulum_Post_L | default | 4.4 |
| Cerebelum_8_L | cerebellum | Temporal_Inf_R | default | 4.4 |
| Vermis_10 | cerebellum | Cerebelum_7b_R | cerebellum | 4.2 |
| Cerebelum_10_L | cerebellum | Cerebelum_8_R | cerebellum | 4.4 |
| Vermis_10 | cerebellum | Vermis_7 | cerebellum | 4.3 |
| SupraMarginal_L | sensorimotor | Calcarine_L | occipital | -3.3 |
| SupraMarginal_L | sensorimotor | Cuneus_L | occipital | -3.6 |
| Postcentral_R | sensorimotor | Lingual_R | occipital | -3.0 |
| SupraMarginal_L | sensorimotor | Lingual_R | occipital | -4.3 |
| SupraMarginal_L | sensorimotor | Occipital_Sup_L | occipital | -3.6 |
| SupraMarginal_L | sensorimotor | Frontal_Mid_Orb_L | fronto-parietal | -3.0 |
| Heschl_L | sensorimotor | Supp_Motor_Area_L | fronto-parietal | -3.0 |
| Temporal_Sup_R | sensorimotor | Parietal_Inf_L | fronto-parietal | -4.0 |
| Temporal_Sup_R | sensorimotor | Parietal_Inf_R | fronto-parietal | -3.4 |
| **Region label A** | **Network A** | **Region label B** | **Network B** | **Bootstrap ratio** |
| Paracentral_Lobule_R | sensorimotor | Angular_R | fronto-parietal | -3.3 |
| Postcentral_R | sensorimotor | Cingulum_Post_R | default | -3.4 |
| Paracentral_Lobule_R | sensorimotor | Precuneus_L | default | -3.0 |
| Occipital_Inf_L | occipital | Calcarine_L | occipital | -3.4 |
| Lingual_R | occipital | Cuneus_L | occipital | -3.2 |
| Occipital_Sup_R | occipital | Frontal_Mid_L | fronto-parietal | -3.2 |
| Occipital_Inf_R | occipital | Supp_Motor_Area_L | fronto-parietal | -3.4 |
| Fusiform_L | occipital | Supp_Motor_Area_L | fronto-parietal | -3.7 |
| Cuneus_R | occipital | Precentral_L | default | -3.2 |
| Calcarine_R | occipital | Precentral_R | default | -3.3 |
| Cuneus_R | occipital | Precentral_R | default | -3.1 |
| Occipital_Sup_L | occipital | Precentral_R | default | -3.0 |
| Occipital_Sup_R | occipital | Precentral_R | default | -3.0 |
| Occipital_Sup_L | occipital | Caudate_R | cingulo-opercular | -3.3 |
| Occipital_Sup_L | occipital | Amygdala_L | cingulo-opercular | -3.4 |
| Parietal_Inf_R | fronto-parietal | Cuneus_L | occipital | -4.0 |
| Parietal_Inf_R | fronto-parietal | Lingual_R | occipital | -3.2 |
| Parietal_Inf_R | fronto-parietal | Occipital_Sup_L | occipital | -4.0 |
| Parietal_Inf_R | fronto-parietal | Rectus_L | default | -3.9 |
| Parietal_Inf_R | fronto-parietal | Rectus_R | default | -3.2 |
| Parietal_Inf_R | fronto-parietal | Cingulum_Post_R | default | -3.0 |
| Precuneus_R | default | Calcarine_L | occipital | -4.2 |
| Precuneus_R | default | Cuneus_L | occipital | -3.3 |
| Precuneus_L | default | Lingual_R | occipital | -3.2 |
| Precuneus_R | default | Lingual_R | occipital | -3.8 |
| Precuneus_R | default | Occipital_Sup_L | occipital | -3.4 |
| Frontal_Sup_Medial_R | default | Supp_Motor_Area_L | fronto-parietal | -3.4 |
| Precuneus_L | default | Cingulum_Post_L | default | -3.2 |
| Putamen_R | cingulo-opercular | Cuneus_L | occipital | -3.3 |
| ParaHippocampal_L | cingulo-opercular | Frontal_Mid_L | fronto-parietal | -3.3 |
| Caudate_L | cingulo-opercular | Parietal_Inf_L | fronto-parietal | -3.2 |
| Hippocampus_L | cingulo-opercular | Precentral_L | default | -4.0 |
| Hippocampus_L | cingulo-opercular | Precentral_R | default | -3.7 |
| Thalamus_L | cingulo-opercular | Caudate_R | cingulo-opercular | -3.3 |
| Cerebelum_Crus1_R | cerebellum | Postcentral_L | sensorimotor | -3.4 |
| Cerebelum_Crus2_R | cerebellum | Postcentral_L | sensorimotor | -3.4 |
| Cerebelum_Crus2_L | cerebellum | Heschl_L | sensorimotor | -3.3 |
| Cerebelum_6_R | cerebellum | Cuneus_L | occipital | -3.0 |
| Vermis_10 | cerebellum | Frontal_Mid_L | fronto-parietal | -3.0 |
| **Region label A** | **Network A** | **Region label B** | **Network B** | **Bootstrap ratio** |
| Cerebelum_4_5_R | cerebellum | Supp_Motor_Area_L | fronto-parietal | -4.6 |
| Cerebelum_Crus1_R | cerebellum | Parietal_Inf_L | fronto-parietal | -3.0 |
| Cerebelum_Crus1_L | cerebellum | Angular_R | fronto-parietal | -3.2 |
| Cerebelum_Crus1_R | cerebellum | Precuneus_L | default | -4.0 |
| Cerebelum_Crus2_R | cerebellum | Precuneus_R | default | -3.7 |
| Cerebelum_Crus1_R | cerebellum | Cingulum_Ant_L | default | -3.7 |
| Cerebelum_Crus2_R | cerebellum | Cingulum_Ant_R | default | -3.4 |
| Cerebelum_Crus2_L | cerebellum | Frontal_Mid_Orb_R | default | -3.2 |
| Cerebelum_6_R | cerebellum | Cingulum_Post_R | default | -3.2 |
| Cerebelum_10_L | cerebellum | Cingulum_Post_R | default | -3.2 |
| Cerebelum_Crus2_L | cerebellum | Cingulum_Mid_R | cingulo-opercular | -3.3 |
| Cerebelum_4_5_R | cerebellum | Caudate_R | cingulo-opercular | -3.4 |
| Cerebelum_9_L | cerebellum | Caudate_R | cingulo-opercular | -3.0 |
| Cerebelum_Crus2_R | cerebellum | Putamen_L | cingulo-opercular | -4.6 |
| Cerebelum_8_L | cerebellum | Pallidum_R | cingulo-opercular | -3.6 |
| Cerebelum_7b_R | cerebellum | Temporal_Pole_Mid_L | cingulo-opercular | -3.0 |
| Vermis_4_5 | cerebellum | Temporal_Pole_Mid_R | cingulo-opercular | -3.0 |
| Cerebelum_6_R | cerebellum | Cerebelum_Crus1_L | cerebellum | -3.2 |
| Cerebelum_10_L | cerebellum | Cerebelum_Crus1_L | cerebellum | -4.2 |
| Cerebelum_6_R | cerebellum | Cerebelum_Crus2_L | cerebellum | -3.5 |
| Cerebelum_10_R | cerebellum | Cerebelum_3_L | cerebellum | -3.6 |
| Cerebelum_7b_R | cerebellum | Cerebelum_3_R | cerebellum | -3.8 |
| Cerebelum_8_L | cerebellum | Cerebelum_4_5_L | cerebellum | -3.5 |
